# In vitro hip testing in the International Society of Biomechanics coordinate system

**DOI:** 10.1016/j.jbiomech.2016.10.036

**Published:** 2016-12-08

**Authors:** Richard J. van Arkel, Jonathan R.T. Jeffers

**Affiliations:** Department of Mechanical Engineering, Imperial College London, London SW7 2AZ, United Kingdom

**Keywords:** Hip, In vitro, Bone pots, Coordinate system, Reference frame

## Abstract

Many innovative experiments are designed to answer research questions about hip biomechanics, however many fail to define a coordinate system. This makes comparisons between studies unreliable and is an unnecessary hurdle in extrapolating experimental results to clinical reality. The aim of this study was to present a specimen mounting protocol which aligns and registers hip specimens in the International Society of Biomechanics (ISB) coordinate system, which is defined by bony landmarks that are identified by palpation of the patient׳s body. This would enable direct comparison between experimental testing and clinical gait analysis or radiographic studies. To represent the intact hip, four intact synthetic full-pelves with 8 full-length articulating femora were assembled and digitised to define the ISB coordinate system. Using our proposed protocol, pelvis specimens were bisected into left and right hemi-pelves and femora transected at the mid-shaft, and then mounted in bone pots to represent a typical experimental setup. Anatomical landmarks were re-digitised relative to mechanical features of the bone pots and the misalignment was calculated. The mean misalignment was found to be less than 1.5° flexion/extension, ab/adduction and internal/external rotation for both the pelves and femora; this equates to less than 2.5% of a normal range of hip motion. The proposed specimen mounting protocol provides a simple method to align in vitro hip specimens in the ISB coordinate system which enables improved comparison between laboratory testing and clinical studies. Engineering drawings are provided to allow others to replicate the simple fixtures used in the protocol.

## 1 Introduction

In the past 10 years, many research labs have developed new methods to study hip joint biomechanics including: digital image correlation (Dickinson et al., 2012, Dickinson et al., 2011), roentgen stereophotogrammetric analysis (Dy et al., 2008, Myers et al., 2011), digital variable resistance transducers (Safran et al., 2011, Smith et al., 2011), real-time contact-pressure measurement (Lee et al., 2015, Rudert et al., 2014), fluid infusion devices (Cadet et al., 2012, Dwyer et al., 2014), optical tracking motion analysis (Lopomo et al., 2010, Signorelli et al., 2013), 3D digital reconstructions combining CT scans and motion tracking (Dwyer et al., 2014, Incavo et al., 2011), combined use of in-vitro and finite element modelling (Anderson et al., 2008, Dickinson et al., 2011, Elkins et al., 2011), custom built rigs in servo-hydraulic actuators/materials testing machines (Dickinson et al., 2012, Elkins et al., 2011, Ito et al., 2009, Song et al., 2012, van Arkel et al., 2015a, van Arkel et al., 2015b) and six-degrees-of-freedom robotic load/torque actuators (Colbrunn et al., 2013, Smith et al., 2014). Such variation in testing methodology not only allows new hypotheses to be tested but also prevents systematic bias that could result from using the same methodology with the same limitations. However, to compare experiments, results need to be reported in a well-defined coordinate system, and to compare to the clinical scenario, it could be advantageous that testing is performed in a clinically adopted coordinate system. Many research studies, including many of those mentioned above, fail to report or reference a full coordinate system; most commonly, the body reference frames for the pelvis and/or femur are under-defined. This is not a recent problem: two decades ago, an extensive critical review of in-vitro testing methods for studying hip prosthesis found that 95% of studies did not fully define a reference frame for the femur (Cristofolini, 1997).

The ISB have published a well-defined hip coordinate system based on the hip centre of rotation, anterior and posterior superior iliac spines (ASIS and PSIS) and femoral epicondyles (Wu et al., 2002). These landmarks are easy to identify non-invasively and consequently have been widely adopted in gait analysis and related musculoskeletal modelling research. Whilst the coordinate systems would be equally beneficial when testing in-vitro, they can be challenging to implement and are rarely used. For example, identifying the femoral head centre is challenging in-vitro and a full pelvis/femur (to identify the ACIS/PSIS and femoral epicondyles) is commonly too large for the available working volume of test rigs or materials-testing-machines. Indeed, most authors test with only hemi-pelves or proximal femora preventing use of the ISB or equivalent system (Anderson et al., 2008, Colbrunn et al., 2013, Crawford et al., 2007, Dickinson et al., 2012, Dickinson et al., 2011, Dwyer et al., 2014, Dy et al., 2008, Elkins et al., 2011, Ito et al., 2009, Lee et al., 2015, Myers et al., 2011, Rudert et al., 2014, Smith et al., 2014, Smith et al., 2011, Song et al., 2012). These authors use pots to fix the bones with varying shapes into engineered testing rigs. The specimens are typically secured into the pot with putty/cement and/or bolts/screws whilst the pots have regular/machined features to attach them in a repeatable manner to the testing rig. Whilst standardising testing rigs would unnecessarily limit experimental methodology, a standardised method to orientate bones into pots whilst maintaining the ISB body reference frames would be beneficial.

Thus, the aim of this study is to provide a method to register the ISB body reference frames to bones before bisecting the pelvis and transecting the femur, and then restore the same coordinate system when the specimen is installed in the experimental fixtures. This would enable in vitro testing to be performed in the same coordinate system as clinical studies and allow greater comparison between in vitro and in vivo work.

## 2 Materials and method

8 solid foam femora and 4 solid foam pelves, 2 each of male/female left/right hemipelvis/femora (Sawbone AB, Sweden, model numbers: #1120, #1120-20, #1129, #1129-21, #1301, #1302) were used in the study. Each pelvis was assembled with two femora and both hip joints were covered with an artificial hip capsule (a paper sleeve covering and encasing the femoral head and neck) to prevent direct visualisation of the femoral head. For each bone model, Ø3.5×10 mm screws were inserted into anatomical landmarks as detailed in Table 1. The crossheads of these screws provide a repeatable point for a Polaris optical tracking system׳s (Northern Digital Inc., Ontario, Canada) digital probe. The screw positions allowed for the ISB body reference frames for the pelvis/femur to be digitised as well as providing seven repeatable points that would be available for re-calculating the pose of the bones after potting them. Whilst three repeatable points per bone would be needed mathematically for subsequent pose estimation calculations, seven were used with as larger spatial distribution as possible to improve accuracy (Challis, 1995). All screws were digitised using the optical tracking system three times. Between repeats the bones were re-orientated in the field of view of the optical tracking system to prevent systematic point registration errors.

**Fig. 1:**
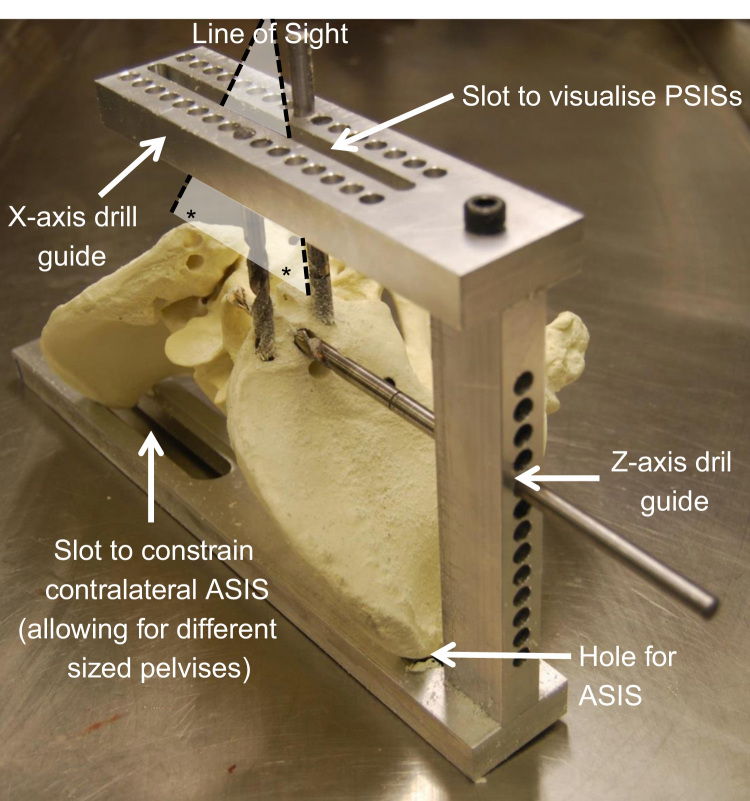
Pelvis drilling guide (femora not shown for clarity). The anterior superior iliac spines (ASISs) are first located in the bottom hole and slot. The pelvis is then rotated until the posterior superior iliac spines (PSISs) can be visualised through the top slot. Holes representing the ISB X and Z axes can then be drilled into the pelvis using the guide.

**Fig. 2:**
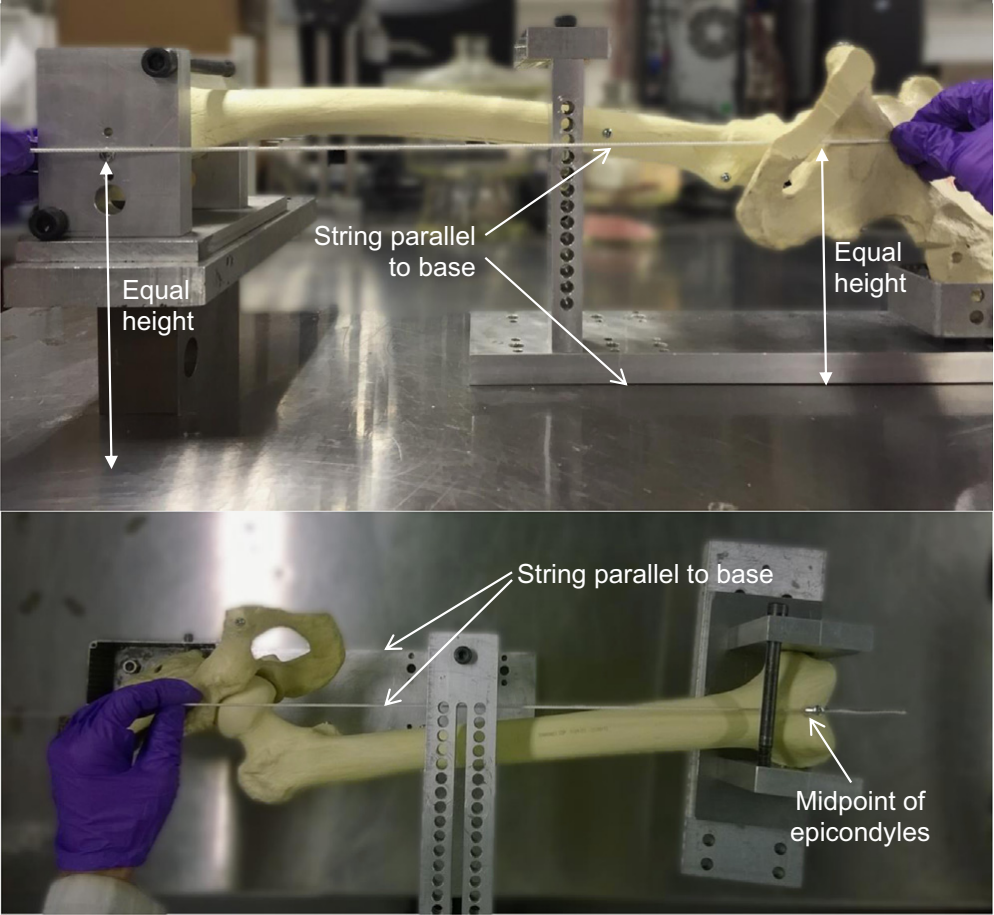
Femoral drilling guide (the artificial capsule has been removed for clarity). The epicondyles are clamped in the middle of two equal sized plates to set neutral rotation when placed on a horizontal surface. The epicondyles are then moved in first the sagittal plane in a movement akin to flexion/extension (top), then in the coronal plane in a movement akin to ab/adduction (bottom) until the femoral y-axis aligns with the length of the drilling jig in both planes. Holes representing the ISB x and z axes can then be drilled into the femoral shaft. The femur can be supported by using a potted hemipelvis, as shown, or in the absence of the pelvis by supporting the femoral head directly.

**Fig. 3:**
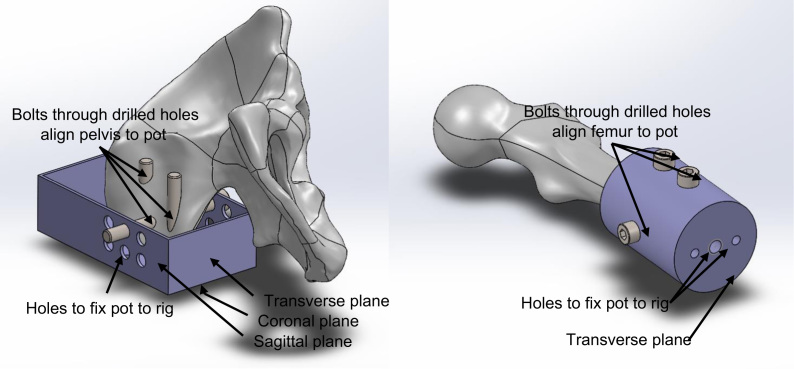
Example pot designs showing how the drilled holes were used to align the bones to their pots. The critical design consideration for alternative pot designs concerns the three bolts passing through these holes: they need to align with machined features of the pot, such as a flat plane and/or fixation holes. This is so that the drilled holes (which preserve the bone׳s reference frame) can be aligned with the axes of the testing rig via the pot.

The hips were prepared with the drilling guides, with the head centre estimated by manual palpation through the artificial capsule (Fig. 1, Fig. 2 and Supplementary material) before bisecting the pelves and transecting the femora at the mid-shaft. The artificial capsule was removed and the prepared bones were orientated and fixed into the bone pots using the holes drilled in the bones (Fig. 3). The example pots used for this validation were designed to fit an existing set-up (van Arkel et al., 2015a, van Arkel et al., 2015b) and have been described in detail along with a potting procedure in Supplementary material. Mechanical features on these bone pots which fix and orientate the pot to the testing rig (such as the flat faces representing the sagittal/coronal/transverse planes, see Fig. 3) were then digitised with the optical tracking probe before the seven repeatable points on each of the bones were re-digitised. Again this was repeated three times rotating the potted specimen in the optical tracker׳s field of view between.

### 2.1 Data analysis

For each repeat of each intact femur/pelvis, the ISB-body reference frame was defined as a matrix of x, y, and z unit vectors,TISBG, in MatLab (version 2011b, The MathWorks, Inc., Texas, USA). The seven repeatable points were transformed into this ISB-body frame using Eq. (1) (Shabana, 2013) and then averaged across the three repeats.

(1) [pISB]=[TISBG]−1[pG]

where pG and pISB are the digitised points in the global (optical tracker׳s) and ISB reference frame, respectively. The same procedure was then performed for the potted specimens: first, the pot׳s reference frame (based on the pot׳s mechanical features) was defined as a matrix of unit vectors. Then the seven repeatable landmarks were transformed into the pot׳s reference frame using Eq. (1) before averaging across repeats.

The misalignment of each body was then quantified as the hip joint rotation away from the ISB neutral position that would result in the bones new pose relative to the pot׳s metal features; if perfectly mounted in the pot, these angles would be zero and the pot׳s reference frame would be equivalent to the ISB body reference frame. The hip joint rotation matrix, R, was calculated from the seven digitised points by minimising the least squares loss term detailed in Eq. (2) using a singular value decomposition technique (Challis, 1995).

(2) $$\sum_{i=1}^{7}{\left\|q_{i}-\left[R\right]p_{i}-d\right\|}^{2}$$

where $p_{i}$ and $q_{i}$ represent the *i*th points measured the ISB and pot׳s reference frame, respectively, and d a translation vector. Angles of flexion/adduction/rotation according the ISB definition (Wu et al., 2002) were then calculated from R using well-established gait analysis equations (Cappozzo et al., 2005). For femoral misalignment calculations, the pelvic body reference frame was assumed stationary, and vice-versa. A detailed description of the calculation steps can be found elsewhere (Cappozzo et al., 2005, Challis, 1995, van Arkel, 2015).

### 2.2 Statistical analysis

The accuracy and precision of the optical tracking method was evaluated by comparing intact specimen repeats (see Supplementary material). An a priori power analysis using this data and GPower 3.0 (Faul et al., 2007) indicated that 8 samples would be sufficient to detect a 1° difference in alignment before and after potting with 80% power. Paired *t*-tests were used to assess if there were any differences between the pose of the pelvic/femoral bodies before and after using the drilling jigs. The significance level was set to *α*=0.05.

## 3 Results

**Fig. 4:**
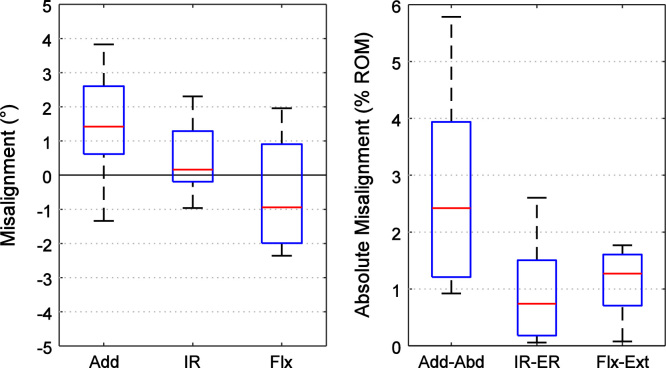
Box plots (*n*=8 specimens, red line=median, blue box=interquartile range, whiskers=range) of the misalignment errors between the ISB and the pot׳s reference frame for the pelvis in terms of adduction/abduction (Add), internal/external rotation (IR), and flexion/extension (Flx). The data is shown in A) degrees and B) the absolute misalignment of the potted pelvis as a percentage of the normal range of hip motion for an adult male (Boone and Azen, 1979). (For interpretation of the references to color in this figure legend, the reader is referred to the web version of this article.)

**Fig. 5:**
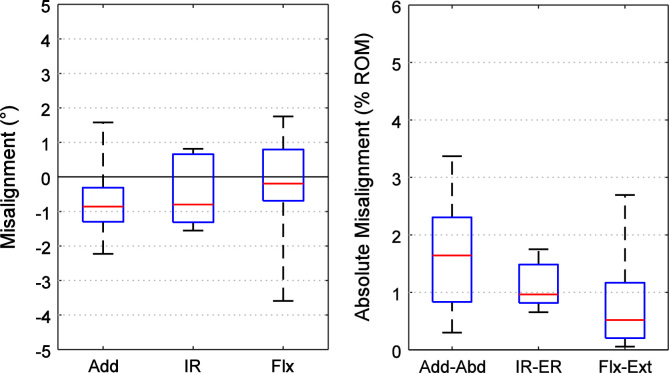
Box plots (*n*=8 specimens, red line=median, blue box=interquartile range, whiskers=range) of the misalignment errors between the ISB and the pot׳s reference frame for the femur in terms of adduction/abduction (Add), internal/external rotation (IR), and flexion/extension (Flx). The data is shown in A) degrees and B) the absolute misalignment of the potted pelvis as a percentage of the normal range of hip motion for an adult male (Boone and Azen, 1979). (For interpretation of the references to color in this figure legend, the reader is referred to the web version of this article.)

The drilling guides could be operated by a single user and added less than half an hour to the potting procedure. For the pelves, the mean±standard deviation misalignment after using the drilling guides to mount the specimens into a bone pot in the ISB pelvic reference frame was: 1.5±1.6° adduction, 0.5±1.1° internal rotation and −0.6±1.7° flexion. For the femoral reference frame, the misalignment was −0.7±1.1° adduction, −0.4±1.0° internal rotation, and 0.4±1.5° flexion. The range of misalignment and the absolute misalignment as a percentage of range of hip motion are shown in Fig. 4, Fig. 5. No differences between the intact and potted specimens were detected except for the pelvis where the specimens were potted in a mean of 1.5° adduction (*p*=0.039).

## 4 Discussion

The most important finding of this study is that low-cost drilling guides can be used to align hip joint specimens into bone pots to enable the use of the ISB body reference frames when performing in-vitro tests with an average misalignment error of 1°. Performing in vitro testing in the ISB coordinate system allows direct comparison between experimental findings and clinical or computational musculoskeletal studies that commonly use this reference frame.

For cadaveric testing, some authors recommend alternative coordinate systems to the ISB system based on anatomical landmarks that have the smallest inter-specimen variance (Cristofolini, 1997, Yoshioka et al., 1987). Other researchers have suggested alternative reference frames for the pelvis relying on the mid-sagittal plane at the pubis symphysis when only a hemipelvis is available (Delp et al., 1999). It is envisaged that adaptions to the drilling guides could be made to implement these alternative coordinate systems if required. However, these coordinate systems are suitable for in vitro testing, but cannot be used in clinical work due to the landmarks not being available. Thus transformation of coordinate systems would be required to compare to clinical work. Custom alignment fixtures/guides have been mentioned in numerous studies and are likely equally effective as the system presented here (Dy et al., 2008, Smith et al., 2014, Song et al., 2012), however they are not described in detail or accompanied by a quantitative validation of their repeatability preventing other users from replicating them. Anderson et al. also used an optical tracking method to iteratively align specimens to within ±1° of the desired orientation (Anderson et al., 2008), similar to the accuracy achieved by the drilling guides. Verstraete et al. report an innovative CT/3D printing based system for alignment of knee joints in vitro and report an accuracy of 3–4° whilst preserving all soft tissues about the knee (which typically increase misalignment) (Verstraete et al., 2016). Other authors have also developed methods for using motion tracking systems to test hip joint specimens in a known coordinate system including: directly using optical tracking systems during experiments (Lopomo et al., 2010, Safran et al., 2013, Signorelli et al., 2013), co-registering CT scans with in-vitro infra-red tracking data (Crawford et al., 2007, Dwyer et al., 2014, Incavo et al., 2011), or programming digitised points into robotic systems (Colbrunn et al., 2013). These methods can offer more scope for data analysis, but are not appropriate for all tests as they require line of sight and/or access to specialist equipment (the optical tracking was only used in the present study to measure the accuracy of the mounting protocol).

**Fig. 6:**
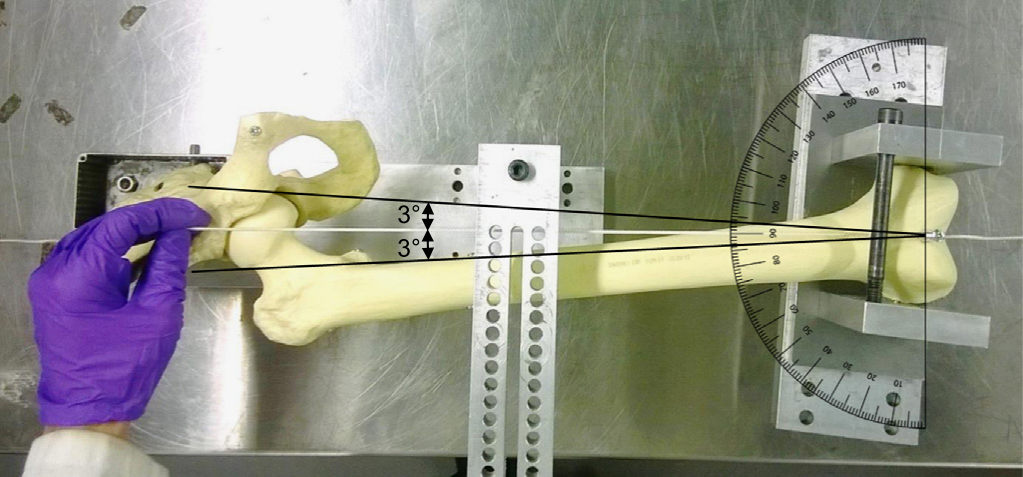
Photograph of the possible error in locating the centre of the femoral head in the coronal plane. The white string highlights the femoral y-axis. The black lines extend from the midpoint of the epicondyles to the medial and lateral boundaries of the femoral head and thus represent the maximum error possible for the mechanical axis assuming the user can palpate/locate any point on the femoral head. This demonstrates how even large errors in identifying the femoral head centre result in relatively small angular misalignment of the femoral y-axis (<3°) due to the long length of the femur. The same concept applies to errors when locating the femoral head centre in the sagittal plane.

The greatest errors when using the drilling jig method were measured in adduction for the pelvis (mean 1.5°, *p*=0.039). Whilst this difference was detected statistically, it is similar in magnitude to that reported by others (Anderson et al., 2008, Verstraete et al., 2016) and is small compared to a typical clinical range of motion (the worst case error recorded for any one specimen was less than 6% of the total range of motion). The error was likely caused by the steep angle of the pelvis when drilling adjacent to the PSIS leading to small deflections of the drill bit. This could perhaps be improved by using deeper holes for the drill guides or by using a larger diameter (and hence stiffer) drill bit; though there is an inherent trade-off between increasing hole size and preserving bone for fixation. For the femur, where the mid-line of the shaft presents a flatter surface, the misalignments were smaller with no differences detected (all *p*>0.12). The validation was carried out by a single user on synthetic bones and not cadaveric tissue which could have introduced three sources of error: firstly, the samples were limited to the anatomy of only one male and female which were interpreted by only a single observer. However, by basing the method around the ISB system which relies on common and well defined landmarks, we minimise the risk that anatomical variations (and their interpretation) will impact on the methodology. Secondly, drilled holes in cadaveric tissue with low bone quality could enlarge or cause bone fractures during testing, especially when high loads are applied. Consequently when testing cadaveric specimens (van Arkel et al., 2015a, van Arkel et al., 2015b), we additionally fill the pot with bone cement. Finally, when testing cadaveric specimens that have not been skeletonised, soft-tissue artefacts could affect the implementation of the coordinate system and increase alignment errors through false identification of the necessary anatomical landmarks such as the femoral head centre and the epicondyles, as is the case when using the ISB system for gait analysis (Lopomo et al., 2010, Lu and O׳Connor, 1999). Through relying on the long-axis of the femur the drilling guide is relatively insensitive to exact locating of the femoral head centre – identifying any point on the femoral head will typically limit the error to only 3° (Fig. 6, and Fig. S7 in Supplementary material). To attempt to consider this possible cause of error in the validation, the femoral head was covered with an artificial hip capsule whilst using the femoral drilling guides.

In conclusion, the described mounting system provides a repeatable way to align a hemi pelvis/proximal femur in the ISB reference frame without added complexity, time or cost. Engineering drawings and a user guide have been provided in Supplementary material so that the guides and protocol used here can be replicated/improved in other laboratories.

## Conflicts of interest

There are no conflicts of interest.

## Figures and Tables

**Table 1:** Anatomical locations for screw placement.

**Body**	**For ISB Reference Frame**	**Repeatable landmarks (for comparing intact and potted)**
Pelvis	Left anterior superior iliac spine	Anterior superior iliac spine
	Right anterior superior iliac spine	Anterior inferior iliac spine
	Left posterior superior iliac spine	Pubic tubercle
	Right posterior superior iliac spine	Ischial tuberosity
		Posterior acetabular rim
		Superior iliac spine
		Acetabulum centre^a^
		
Femur	Medial femoral epicondyle	Insertion of ligamentum teres
	Lateral femoral epicondyle	Superior tip of greater trochanter
	Femoral head centre^a^	Lateral base of greater trochanter
		Lesser trochanter
		Medial mid-shaft
		Lateral mid-shaft
		Femoral head centre^a^

a These centre points were not pinpointed with screws but were found from a least-squares sphere-fit of >100 digitised points on the surface of the acetabulum/femoral head.
