# Supplementary material for: In vitro hip testing in the International Society of Biomechanics coordinate system
Source: J Biomech. 2016 Dec 8;49(16):4154–8. doi: 10.1016/j.jbiomech.2016.10.036 (PMC5352732; doi:10.1016/j.jbiomech.2016.10.036)
Supplement: Supplementary file 1 — Supplementary material [file mmc1.pdf]

| ITEM NO. | PART NUMBER | DESCRIPTION | QTY. |
|---|---|---|---|
| 1 | JIG03 | PELVIC JIG BASE | 1 |
| 2 | JIG01 | Z-AXIS GUIDE | 1 |
| 3 | FULL-PELVIS | | 1 |
| 4 | JIG02 | X-AXIS GUIDE | 1 |
| 5 | ISO 8734 - 6 x 30 - B | PARALLEL PIN (GROUND TO m6) | 4 |
| 6 | ISO 4762 M8 x 20 | BOTTOM BOLT | 1 |
| 7 | ISO 4762 M8 x 30 | TOP BOLT | 1 |

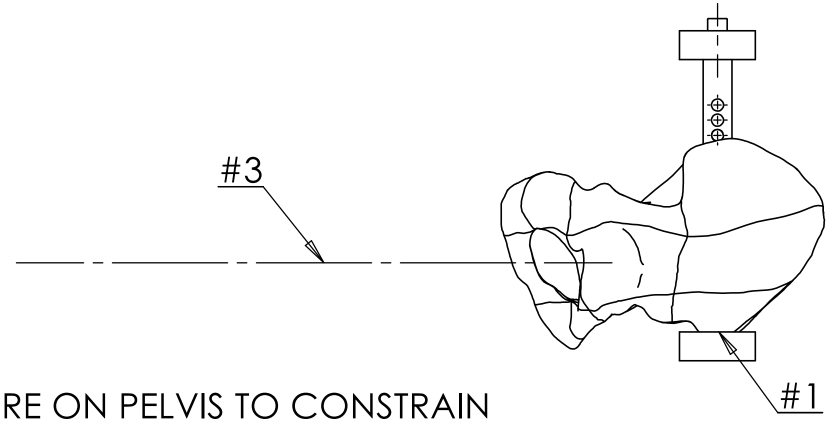


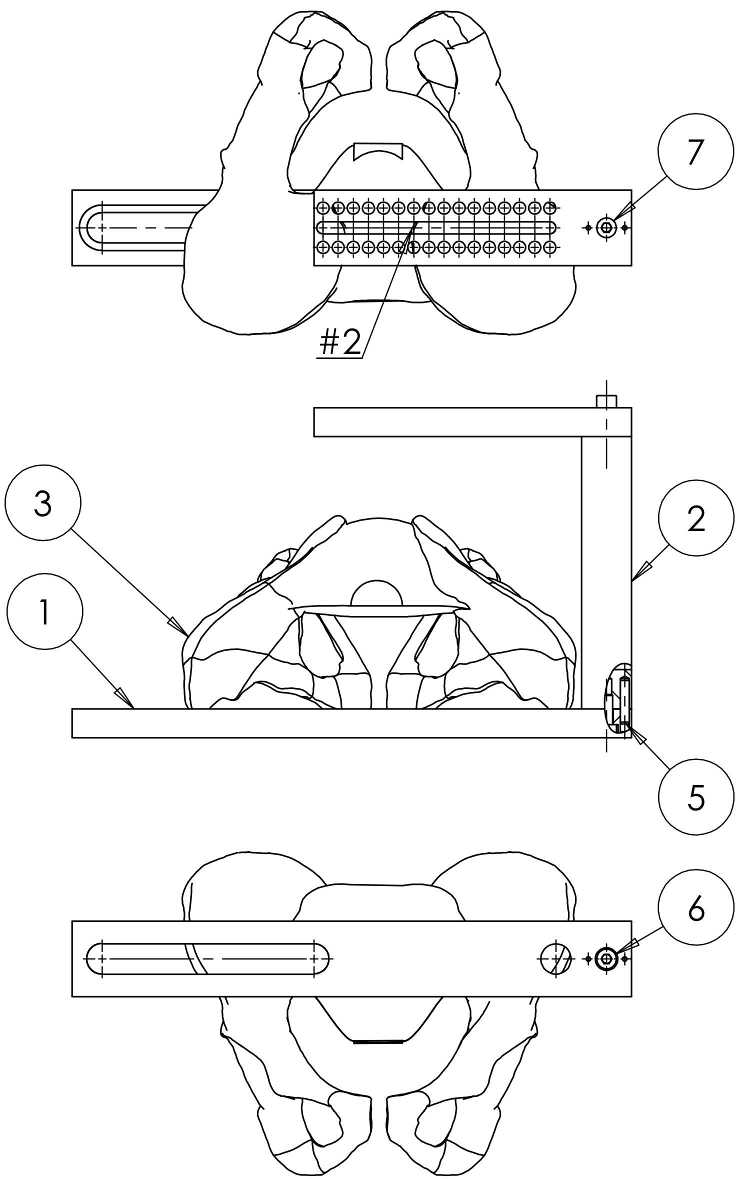


#1 PUT PRESSURE ON PELVIS TO CONSTRAIN ANTERIOR SUPERIOR ILIAC SPINE IN SLOT

#2 ROTATE PELVIS (MOVEMENT AKIN TO FLEXION) UNTIL POSTERIOR SUPERIOR ILIAC SPINE BECOMES VISIBILE IN SLOT

#3 JIG CAN BE USED WITH FULL LENGTH FEMURS ATTACHED WHICH CAN EXTEND DISTALLY IN THIS DIRECTION, NOTE THAT FEMORAL POSITIONING DOES NOT AFFECT THE REFERENCE FRAME OF THE PELVIS.

| TOLERANCES | | THIRD ANGLE PROJECTION | MATERIAL: | TITLE: ASSY01 |
|---|---|---|---|---|
| X =± 0.5<br>X.X =± 0.1<br>X.XX =± 0.02 | ANGULAR ±1°<br>SURFACE FINISH MACHINED FACES Ra 6.3 | | ALL DIMENSIONS ARE IN MILLIMETRES | |
| | NAME | DATE | | |
| DRAWN | RvA | 22/04/13 | DO NOT SCALE DRAWING | DWG No. |
| CHECKED | | | | ASSY01 |
| APPROVED | JJ | 24/04/13 | A4 SCALE 1:5 | |

Imperial College London
Department of Mechanical Engineering

SHEET 1 OF 1 REVISION 1

| ITEM NO. | PART NUMBER | DESCRIPTION | QTY. |
|---|---|---|---|
| 1 | PELVIS | | 1 |
| 2 | JIG04-A | SMALL POT | 1 |
| 3 | ISO - 4036 - M8 | THIN NUT | 5 |
| 4 | PMMA | BONE CEMENT | 1 |
| 5 | ISO 4762 M8 x 40 | X-AXIS BOLTS | 2 |
| 6 | ISO 4762 M8 x 70 | Z-AXIS BOLT | 1 |
| 7 | ISO 4762 M8 x 12 | TEMP BOLTS | 5 |
| 8 | ISO 4762 M8 x 16 | TEMP BOLTS | 2 |
| 9 | ISO - 4034 - M8 | NUT | 4 |
| 10 | JIG04-B | ALTERNATIVE POT | 1 |

- USE PEVLIS MOUNTING JIG TO PREPARE HEMI-PELVIS
- ALL PARTS THAT CONTACT CEMENT SHOULD BE COVERED IN A LIGHT LAYER OF GREASE SO THAT THE PARTS CAN BE REUSED BY DISASSEMBLING THE CEMENTED UNIT (NOTE THAT BOLTS WILL AUTOMATICALLY CREATE A MATING THREAD IN THE BONE CEMENT)
- TIGHTEN ALL NUTS AND FILL UNUSED HOLES WITH TISSUE PAPER OR EQUIVALENT BEFORE FILLING ENTIRE PELVIS POT WITH BONE CEMENT
- THICK AND THIN NUTS (ITEMS 3&9) ARE INTERCHANGEABLE
- FOR ITEM NUMBERS 3, 7 & 9: IF EITHER NUT, OR BOLT CANNOT BE TIGHTENED DUE TO CLASH WITH BONE, EITHER MAKE A SMALL BONE RESECTION OR USE A SMALLER NUT/BOLT, OR OMIT THE NUT/BOLT ENTIRELY.
- REMOVE TEMPORARY BOLTS (ITEMS 7&8) ONCE CEMENT CURED. THE RESULTING HOLES ARE FOR RIG ATTACHMENT.
- FOR LARGER PELVES USE ITEM 10, LARGE PELVIC POT (NOT SHOWN)

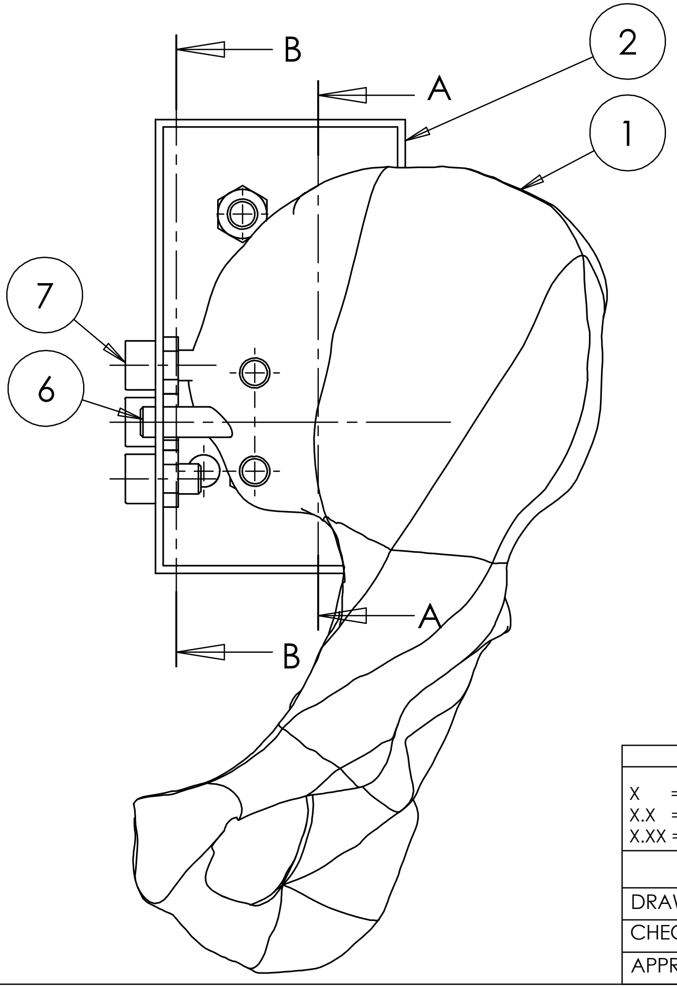


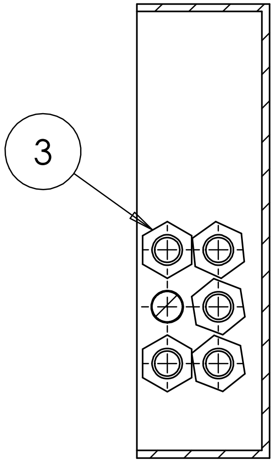


SECTION B-B

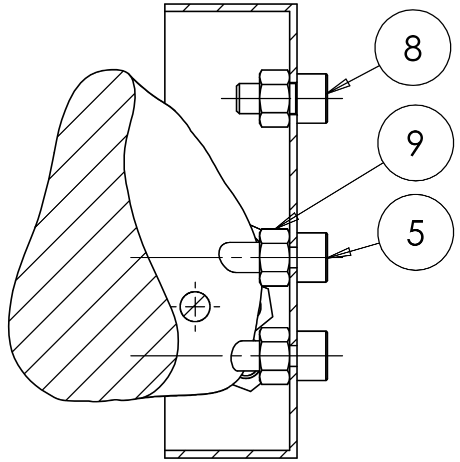


SECTION A-A

| TOLERANCES | | THIRD ANGLE PROJECTION | MATERIAL: | TITLE: |
|---|---|---|---|---|
| X =± 0.5<br>X.X =± 0.1<br>X.XX =± 0.02 | ANGULAR ±1°<br>SURFACE FINISH MACHINED FACES Ra 6.3 | | ALL DIMENSIONS ARE IN MILLIMETRES | POTTED PELVIS |
| | NAME | DATE | | |
| DRAWN | RvA | 22/04/13 | DO NOT SCALE DRAWING | DWG No. |
| CHECKED | | | | |
| APPROVED | JJ | 24/04/13 | A4 SCALE 1:2 | ASSY02 |


Imperial College London
Department of Mechanical Engineering


SHEET 1 OF 1 REVISION 2

| ITEM NO. | PART NUMBER | DESCRIPTION | QTY. |
|---|---|---|---|
| 1 | JIG05 | FEMORAL JIG BASE | 1 |
| 2 | FULL-FEMUR | | 1 |
| 3 | JIG07 | EPICONDYLE CLAMP 2 | 1 |
| 4 | JIG06 | EPICONDYLE CLAMP 1 | 1 |
| 5 | JIG01 | Z-AXIS GUIDE | 1 |
| 6 | JIG02 | Y-AXIS GUIDE | 1 |
| 7 | JIG08 | OPTIONAL SPACER #2 | 2 |
| 8 | ISO 4762 M8 x 50 | PELVIC POT MOUNTING BOLTS | 2 |
| 9 | ISO 4762 M8 x 80 | EPICONDYLE HEIGHT BOLTS | 2 |
| 10 | ISO 4762 M10 x 130 | EPICONDYLE CLAMP BOLTS | 4 |
| 11 | ASSY02 | POTTED PELVIS | 1 |
| 12 | ISO 7049 4.2 x 25 | SELF TAPPING SCREWS | 3 |
| 13 | ISO 4762 M8 x 20 | Z-AXIS BOTTOM BOLT | 1 |
| 14 | ISO 4762 M8 x 30 | Z-AXIS TOP BOLT | 1 |
| 15 | ISO 8734 - 6 x 30 - B | PARALLEL PIN (GROUND TO m6) | 4 |

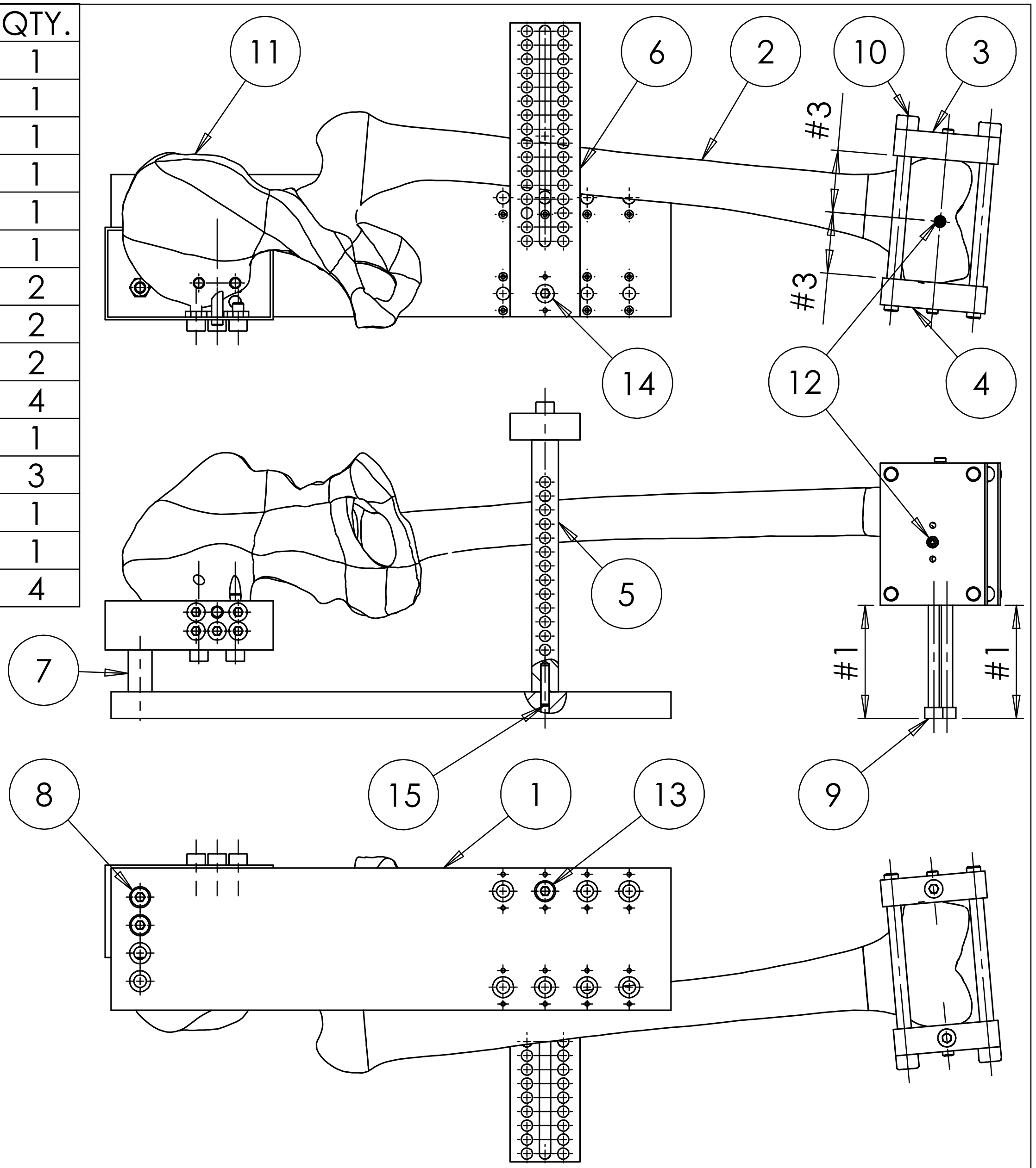


#1 TO SET NEUTRAL ROTATION, FEMORAL EPICONDYLES NEED TO BE PARALLEL TO THE BASE SO DISTANCE #1 NEEDS TO BE THE SAME FOR BOTH BOLTS. ALTERNATIVELY COULD BE RESTED ON SCRAP FLAT METAL BAR/BOX.

#2 THIS SPACER COULD BE REPLACED BY WASHERS OR EQUIVALENT. ITS PURPOSE IS TO SHORTEN THE X-AXIS DRILLING LENGTH TO IMPROVE ACCURACY. NOTE THE ORIENATION OF THE PELVIC POT IS NOT IMPORTANT PROVIDED IT IS RIGIDLY FIXED.

#3 INSERT SELF TAPPING SCREW (ITEM 12) AT THE MID-POINT BETWEEN FEMORAL EPICONDYLES (MEASURE WITH RULE). EXTEND A SUTURE/PIECE OF SRING FROM THIS SCREW TO THE FEMORAL HEAD CENTRE TO ALIGN MECHANICAL AXIS. ONE CLAMP BOLT (ITEM 10) MAY NEED TO BE REMOVED TO ENABLE FREE PASSAGE OF SUTURE/STRING.

| | | |
|---|---|---|
| TOLERANCES | | THIRD ANGLE PROJECTION |
| X =± 0.5<br>X.X =± 0.1<br>X.XX =± 0.02 | ANGULAR ±1°<br>SURFACE FINISH MACHINED FACES Ra 6.3 | |
| | NAME | DATE |
| DRAWN | RvA | 22/04/13 |
| CHECKED | | |
| APPROVED | JJ | 24/04/13 |

MATERIAL:

ALL DIMENSIONS ARE IN MILLIMETRES

DO NOT SCALE DRAWING

A4 SCALE 1:5

TITLE:

FEMORAL JIG

DWG No.

ASSY03


Imperial College London
Department of Mechanical Engineering


SHEET 1 OF 1 REVISION 1

| ITEM NO. | PART NUMBER | DESCRIPTION | QTY. |
| --- | --- | --- | --- |
| 1 | FEMUR | | 1 |
| 2 | JIG09 | FEMORAL POT | 1 |
| 3 | ISO 4762 M8 x 65 | AXES BOLTS | 3 |
| 4 | ISO 4762 M10 x 20 | TEMP BOLT | 1 |
| 5 | PMMA | BONE CEMENT | 1 |

- USE FEMUR MOUNTING JIG TO PREPARE PROXIMAL FEMUR
- ALL PARTS THAT CONTACT CEMENT SHOULD BE COVERED IN A LIGHT LAYER OF GREASE SO THAT THE PARTS CAN BE REUSED BY DISASSEMBLING THE CEMENTED UNIT (NOTE THAT BOLTS WILL AUTOMATICALLY CREATE A MATING THREAD IN THE BONE CEMENT)
- ASSEMBLE ALL BOLTS BEFORE FILLING POT WITH CEMENT
- REMOVE TEMPORARY BOLT (ITEM 4) ONCE CEMENT CURED

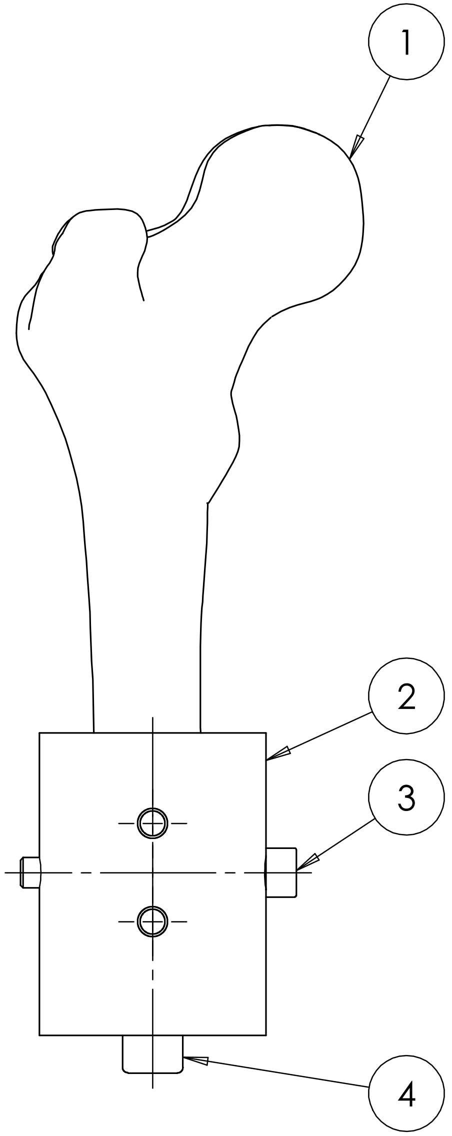


| TOLERANCES | | THIRD ANGLE PROJECTION | MATERIAL: | TITLE: ASSY04 |
| --- | --- | --- | --- | --- |
| X =± 0.5<br>X.X =± 0.1<br>X.XX =± 0.02 | ANGULAR ±1°<br>SURFACE FINISH MACHINED FACES Ra 6.3 | | ALL DIMENSIONS ARE IN MILLIMETRES | |
| | NAME | DATE | | |
| DRAWN | RvA | 22/04/13 | DO NOT SCALE DRAWING | DWG No. ASSY04 |
| CHECKED | | | | |
| APPROVED | JJ | 24/04/13 | A4 / SCALE 1:2 | |

Imperial College London
Department of Mechanical Engineering

SHEET 1 OF 1 | REVISION 1

*ADJUST THICKNESS BASED ON FLAT BAR AVAILABILITY
ANY NON-RUSTING METAL ALLOY FLAT BAR COULD BE USED. FOR EXAMPLE:

- ALLUMINIUM ALLOY 6082T6 (LOWER COST, EASIER MACHINING)
- STAINLESS STEEL 304 (HARDER WEARING)

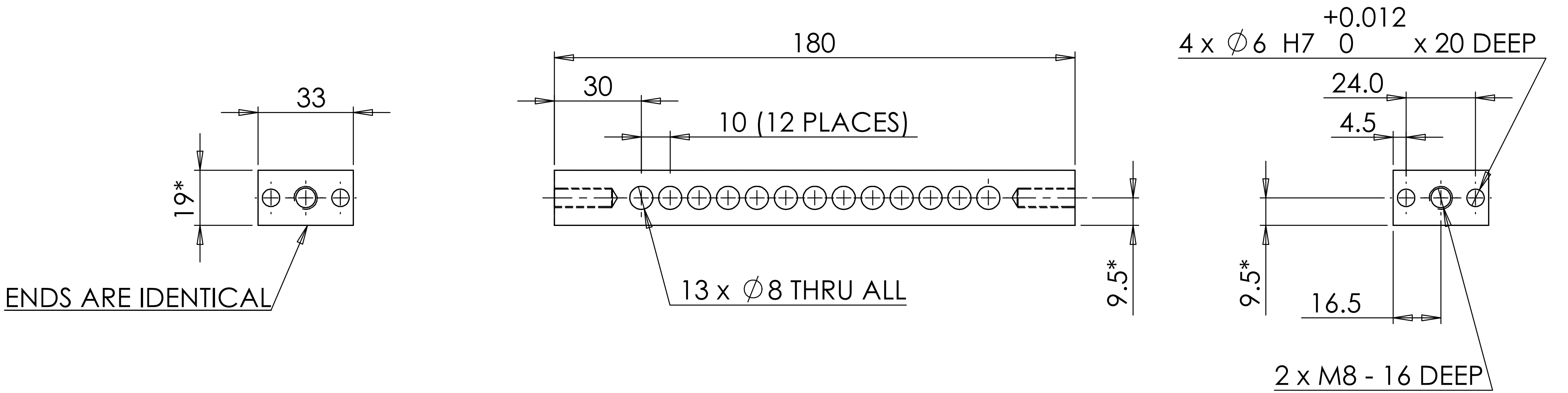


| TOLERANCES | | THIRD ANGLE PROJECTION | MATERIAL: SEE NOTES | TITLE: |
|---|---|---|---|---|
| X =± 0.5<br>X.X =± 0.1<br>X.XX =± 0.02 | ANGULAR ±1°<br>SURFACE FINISH MACHINED FACES Ra 6.3 | | ALL DIMENSIONS ARE IN MILLIMETRES | Z-AXIS GUIDE |
| | NAME | DATE | | |
| DRAWN | RvA | 20/03/13 | DO NOT SCALE DRAWING | DWG No. |
| CHECKED | | | | JIG01 |
| APPROVED | JJ | 27/03/13 | A4 / SCALE 1:2 | |

Imperial College London
Department of Mechanical Engineering

SHEET 1 OF 1 | REVISION 1

NOTES:
#1 HOLES FOR PARALLEL PIN EJECTION

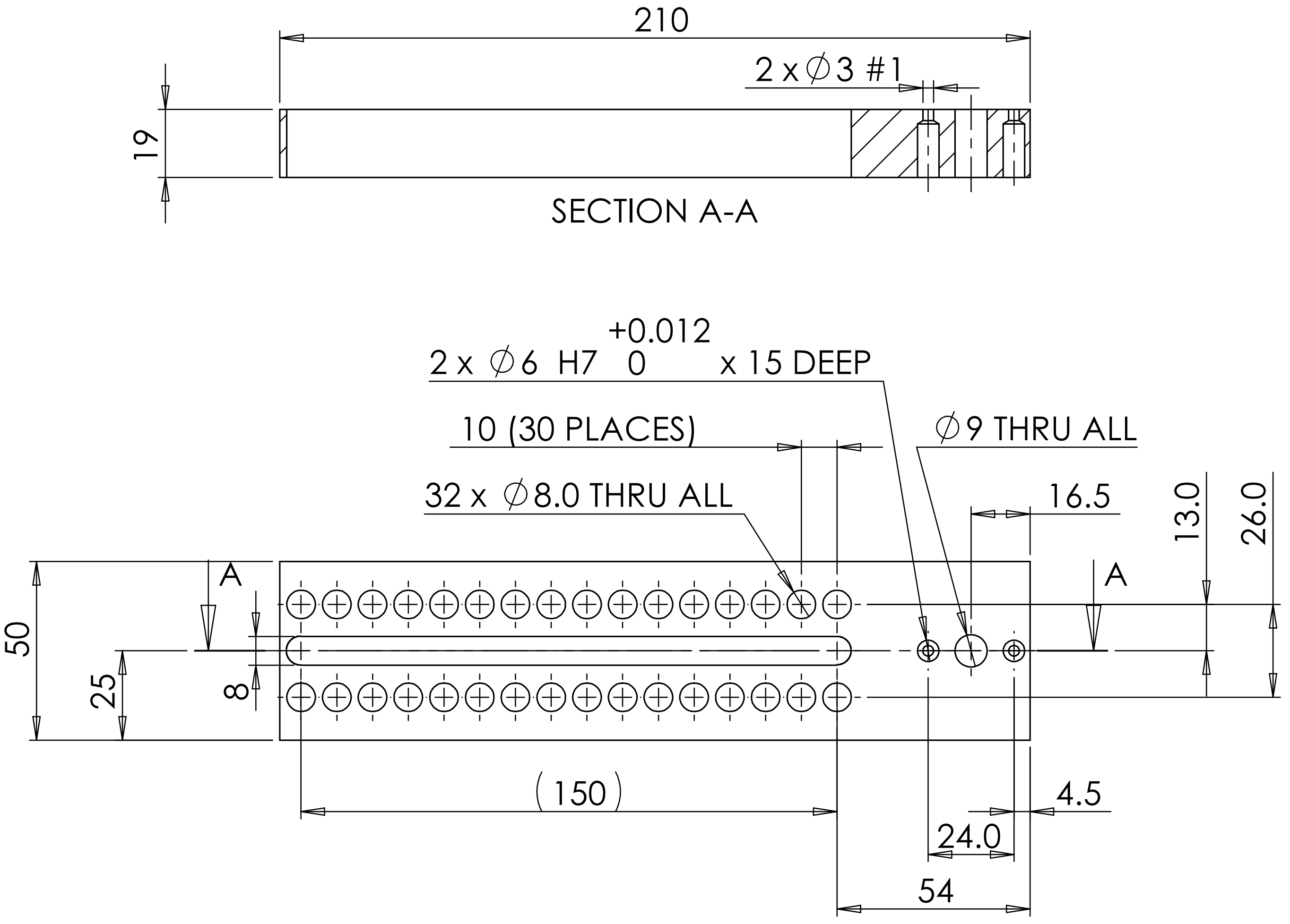


| | | | | | | |
|---|---|---|---|---|---|---|
| TOLERANCES<br>X =± 0.5<br>X.X =± 0.1<br>X.XX =± 0.02 | ANGULAR ±1°<br>SURFACE FINISH MACHINED FACES Ra 6.3 | THIRD ANGLE PROJECTION | MATERIAL:<br>ALUMINIUM ALLOY 6082T6<br>FOR ALTERNATIVE SEE JIG01<br>ALL DIMENSIONS ARE IN MILLIMETRES | TITLE:<br>X-AXIS GUIDE | | Imperial College London<br>Department of Mechanical Engineering |
| | NAME | DATE | | | | |
| DRAWN | RvA | 20/03/13 | DO NOT SCALE DRAWING | DWG No.<br>JIG02 | | |
| CHECKED | | | | | | |
| APPROVED | JJ | 27/03/13 | A4 | SCALE 1:2 | SHEET 1 OF 1 | REVISION 2 |

#1 HOLES FOR PARALLEL PIN EJECTION

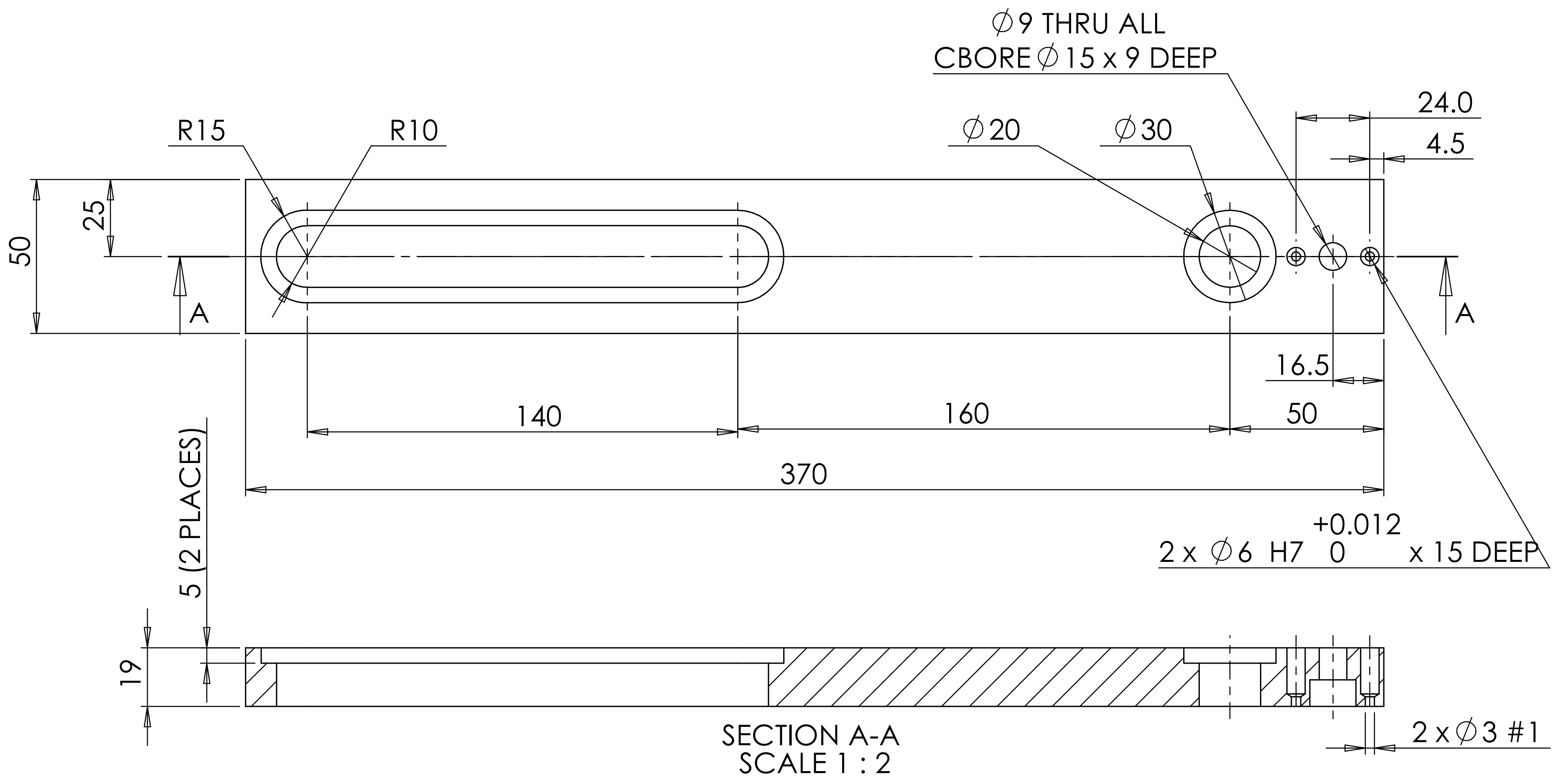


| | | | | | |
|---|---|---|---|---|---|
| TOLERANCES<br>X =± 0.5<br>X.X =± 0.1<br>X.XX =± 0.02 | ANGULAR ±1°<br>SURFACE FINISH MACHINED FACES Ra 6.3 | THIRD ANGLE PROJECTION | MATERIAL:<br>ALUMINIUM ALLOY 6082T6<br>FOR ALTERNATIVE SEE JIG01<br>ALL DIMENSIONS ARE IN MILLIMETRES | TITLE:<br>PELVIC JIG BASE | Imperial College London<br>Department of Mechanical Engineering |
| | NAME | DATE | | | |
| DRAWN | RvA | 20/03/13 | DO NOT SCALE DRAWING | DWG No.<br>JIG03 | |
| CHECKED | | | | | |
| APPROVED | JJ | 27/03/13 | A4 SCALE 1:2 | | SHEET 1 OF 1 REVISION 2 |

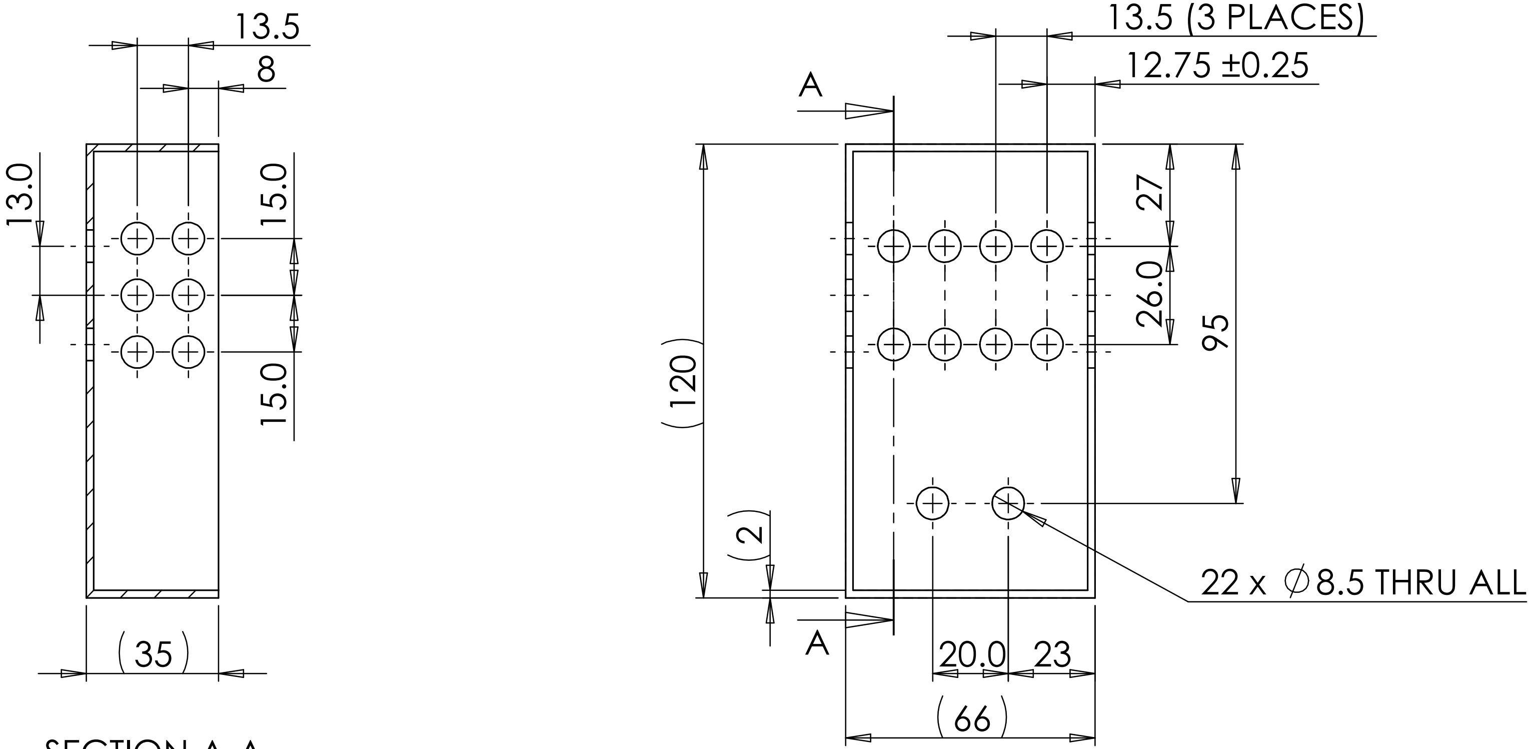


SECTION A-A

| TOLERANCES | | THIRD ANGLE PROJECTION | MATERIAL: ALUMINIUM DIE CAST BOX | TITLE: SMALL PELVIC POT |
|---|---|---|---|---|
| X =± 0.5<br>X.X =± 0.1<br>X.XX =± 0.02 | ANGULAR ±1°<br>SURFACE FINISH MACHINED FACES Ra 6.3 | | ALL DIMENSIONS ARE IN MILLIMETRES | |

| | NAME | DATE |
|---|---|---|
| DRAWN | RvA | 22/04/13 |
| CHECKED | | |
| APPROVED | JJ | 24/04/13 |

DO NOT SCALE DRAWING

A4 | SCALE 1:2

DWG No. JIG04-A

Imperial College London
Department of Mechanical Engineering

SHEET 1 OF 1 | REVISION 1

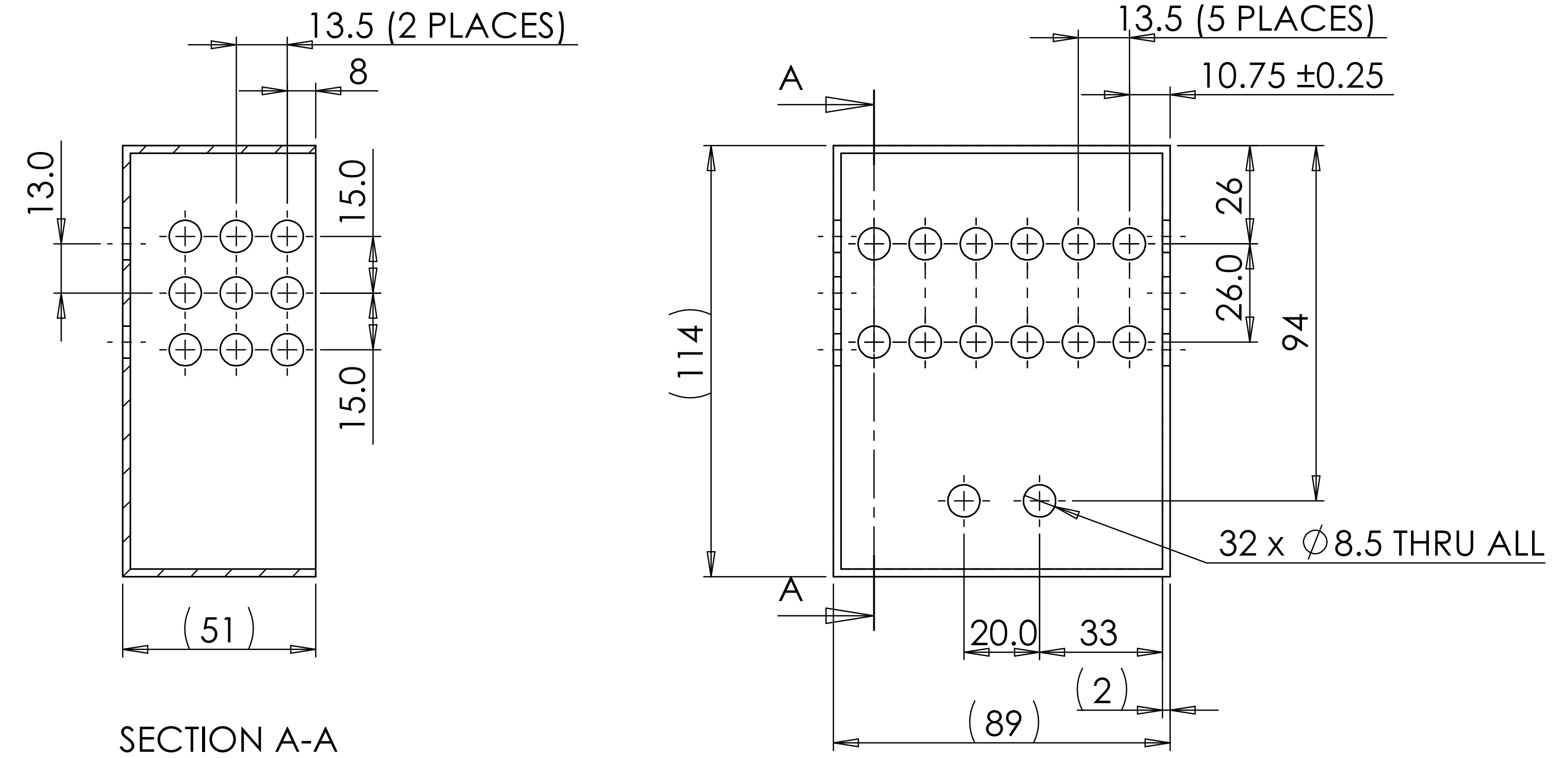


SECTION A-A

| TOLERANCES | | THIRD ANGLE PROJECTION | MATERIAL: ALUMINIUM DIE CAST BOX | TITLE: LARGE PELVIC POT | Imperial College London |
|---|---|---|---|---|---|
| X =± 0.5<br>X.X =± 0.1<br>X.XX =± 0.02 | ANGULAR ±1°<br>SURFACE FINISH MACHINED FACES Ra 6.3 | | ALL DIMENSIONS ARE IN MILLIMETRES | | Department of Mechanical Engineering |
| | NAME | DATE | | | |
| DRAWN | RvA | 22/04/13 | DO NOT SCALE DRAWING | DWG No. JIG04-B | |
| CHECKED | | | A4 SCALE 1:2 | | SHEET 1 OF 1 |
| APPROVED | JJ | 24/04/13 | | | REVISION 1 |

#1 HOLES FOR PARALLEL PIN EJECTION
#2 MACHINE CENTRE LINE (DEPTH 1MM) WITH SPOT DRILL, 90° END MILL, OR EQUIVALENT, SUCH THAT IT IS PERPENDICULAR TO THE LINES CONNECTING THE CENTRE MARKS OF EACH PARALLEL PIN HOLE-PAIR (FOR EXAMPLE DATUM A). THE MECHANICAL AXIS OF THE FEMUR IS ALIGNED AGAINST THIS LINE WHEN USING THE JIG AND HENCE ANY DEVIATIONS FROM PERPENDICULAR WILL BIAS FEMORAL ORIENTATION TO EITHER AB/ADDUCTION. THE GEOMETRIC TOLERANCE GIVEN EQUATES TO A MAXIMUM MISALIGNMENT OF THE FEATURE OF 0.5°

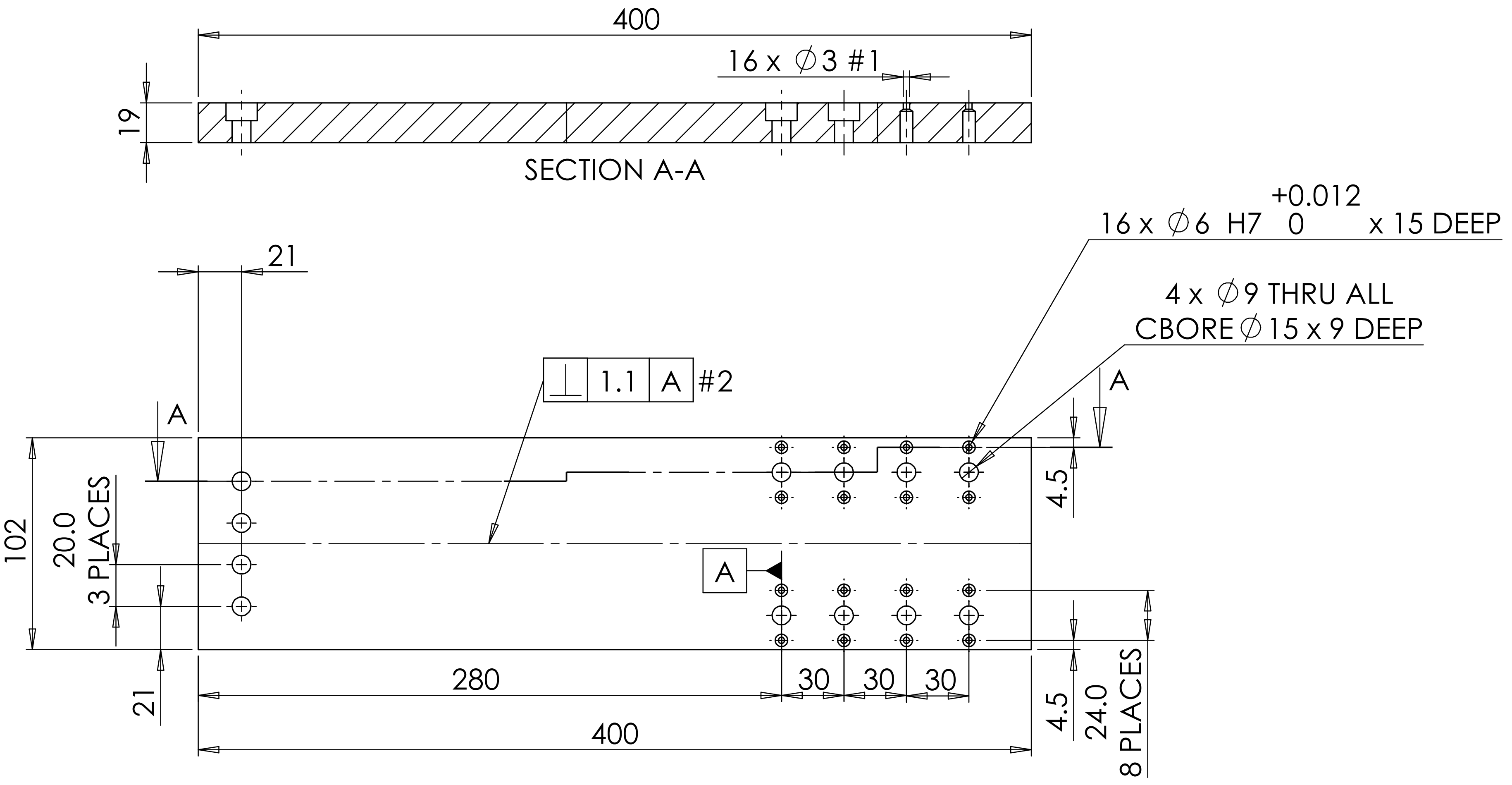


| TOLERANCES | | THIRD ANGLE PROJECTION | MATERIAL: ALUMINIUM ALLOY 6082T6 FOR ALTERNATIVE SEE JIG01 | TITLE: FEMORAL JIG BASE | Imperial College London |
|---|---|---|---|---|---|
| X =± 0.5<br>X.X =± 0.1<br>X.XX =± 0.02 | ANGULAR ±1°<br>SURFACE FINISH MACHINED FACES Ra 6.3 | | ALL DIMENSIONS ARE IN MILLIMETRES | | Department of Mechanical Engineering |
| | NAME | DATE | | | |
| DRAWN | RvA | 16/04/13 | DO NOT SCALE DRAWING | DWG No. | |
| CHECKED | | | | JIG05 | |
| APPROVED | JJ | 24/04/13 | A4 SCALE 1:3 | | SHEET 1 OF 1 REVISION 2 |

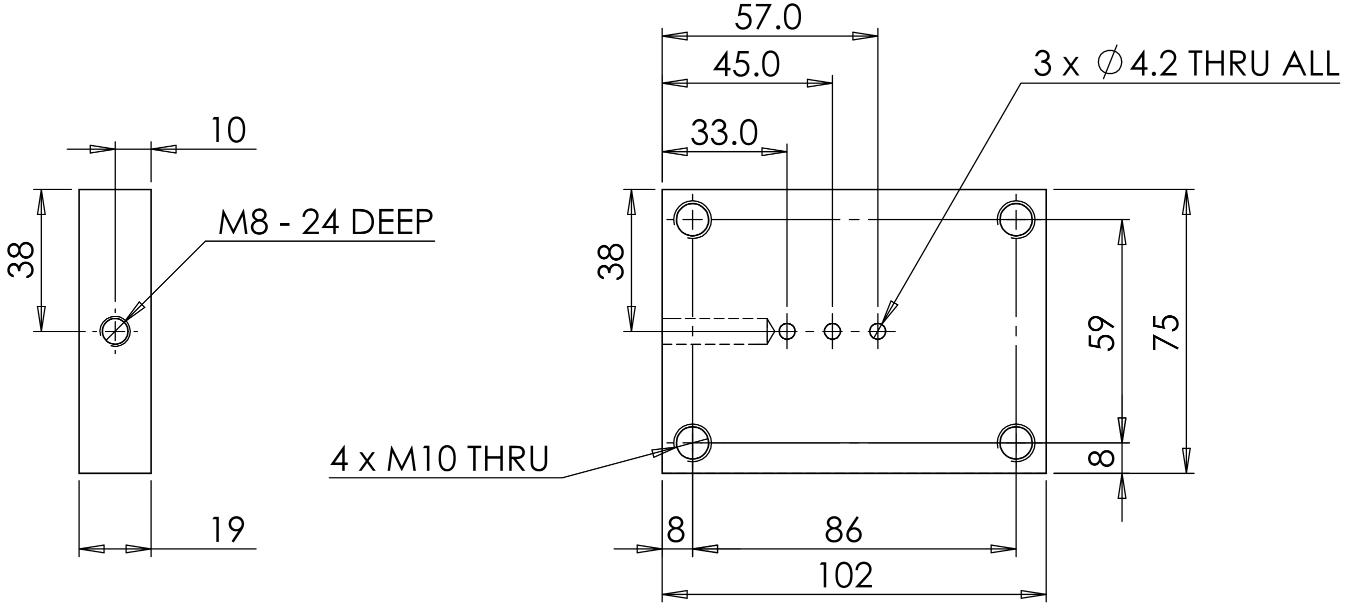


TOLERANCES
X =± 0.5
X.X =± 0.1
X.XX =± 0.02
ANGULAR ±1°
SURFACE FINISH MACHINED FACES Ra 6.3

THIRD ANGLE PROJECTION

MATERIAL:
ALUMINIUM ALLOY 6082T6
FOR ALTERNATIVE SEE JIG01

ALL DIMENSIONS ARE IN MILLIMETRES

| | NAME | DATE |
|---|---|---|
| DRAWN | RvA | 16/04/13 |
| CHECKED | | |
| APPROVED | JJ | 24/04/13 |

DO NOT SCALE DRAWING

A4 SCALE 1:2

TITLE:
EPICONDYLE CLAMP 1

DWG No.
JIG06


Imperial College London
Department of Mechanical Engineering


SHEET 1 OF 1 REVISION 1

IDENTICAL TO JIG06 BUT WITH ∅11 THRU HOLES INSTEAD OF TAPPED HOLES

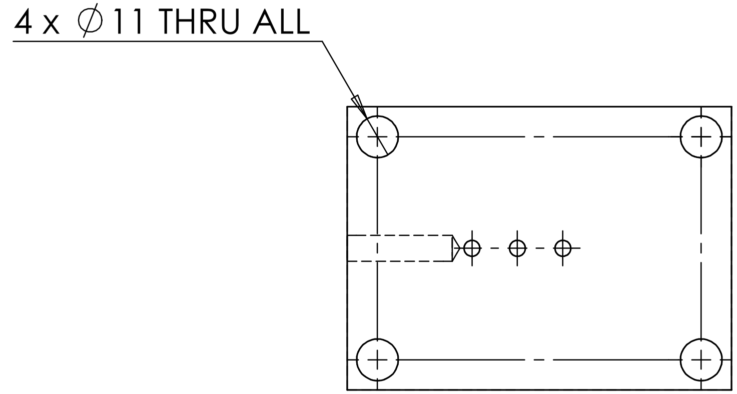


| | | | | |
|---|---|---|---|---|
| TOLERANCES<br>X =± 0.5<br>X.X =± 0.1<br>X.XX =± 0.02 | ANGULAR ±1°<br>SURFACE FINISH MACHINED FACES Ra 6.3 | THIRD ANGLE PROJECTION | MATERIAL:<br>ALUMINIUM ALLOY 6082T6<br>FOR ALTERNATIVE SEE JIG01<br>ALL DIMENSIONS ARE IN MILLIMETRES | TITLE:<br>EPICONDYLE CLAMP 2 |
| | NAME | DATE | DO NOT SCALE DRAWING | DWG No.<br>JIG07 |
| DRAWN | RvA | 16/04/13 | A4 | SCALE 1:2 |
| CHECKED | | | | |
| APPROVED | JJ | 24/04/13 | | |

Imperial College London
Department of Mechanical Engineering

SHEET 1 OF 1 | REVISION 1

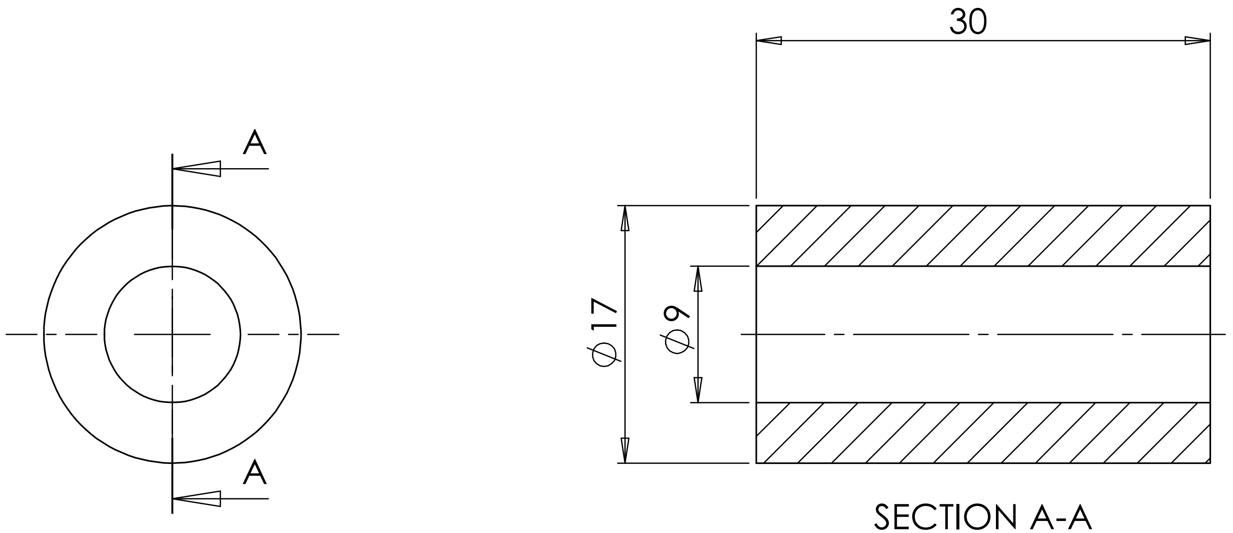


SECTION A-A

| TOLERANCES | | THIRD ANGLE PROJECTION | MATERIAL: ALUMINIUM ALLOY 6082T6 FOR ALTERNATIVE SEE JIG01 | TITLE: SPACER | Imperial College London Department of Mechanical Engineering |
|---|---|---|---|---|---|
| X =± 0.5<br>X.X =± 0.1<br>X.XX =± 0.02 | ANGULAR ±1°<br>SURFACE FINISH MACHINED FACES Ra 6.3 | | ALL DIMENSIONS ARE IN MILLIMETRES | | |
| | NAME | DATE | | | |
| DRAWN | RvA | 22/04/13 | DO NOT SCALE DRAWING | DWG No. JIG08 | |
| CHECKED | | | A4 / SCALE 2:1 | | SHEET 1 OF 1 |
| APPROVED | JJ | 24/04/13 | | | REVISION 1 |

#1 IF MACHINING M8 HOLES AS SEPARATE OPERATION, CLAMP SUCH THAT DATUM B IS VERTICAL TO ENSURE HOLES ARE AS PARALLEL TO BASE AS POSSIBLE

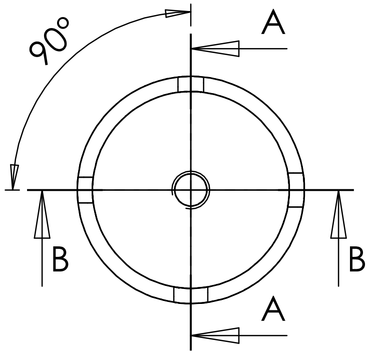


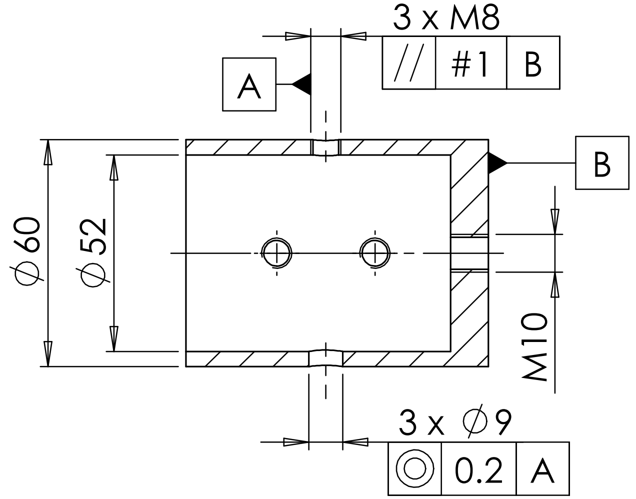


SECTION A-A

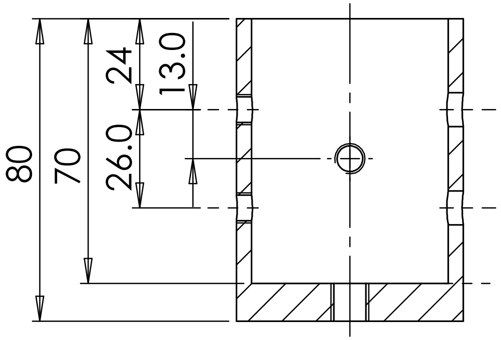


SECTION B-B

| TOLERANCES | | THIRD ANGLE PROJECTION | MATERIAL: ALUMINIUM ALLOY 6082 T6 FOR ALTERNATIVE SEE JIG01 | TITLE: FEMORAL POT | Imperial College London Department of Mechanical Engineering |
|---|---|---|---|---|---|
| X =± 0.5<br>X.X =± 0.1<br>X.XX =± 0.02 | ANGULAR ±1°<br>SURFACE FINISH MACHINED FACES Ra 6.3 | | ALL DIMENSIONS ARE IN MILLIMETRES | | |
| | NAME | DATE | | | |
| DRAWN | RvA | 16/04/13 | DO NOT SCALE DRAWING | DWG No. | |
| CHECKED | | | | | |
| APPROVED | JJ | 24/04/13 | A4 SCALE 1:2 | JIG09 | SHEET 1 OF 1 REVISION 1 |
