# Supplementary material for: In vitro hip testing in the International Society of Biomechanics coordinate system
Source: J Biomech. 2016 Dec 8;49(16):4154–8. doi: 10.1016/j.jbiomech.2016.10.036 (PMC5352732; doi:10.1016/j.jbiomech.2016.10.036)
Supplement: Supplementary file 2 — Supplementary material [file mmc2.pdf]

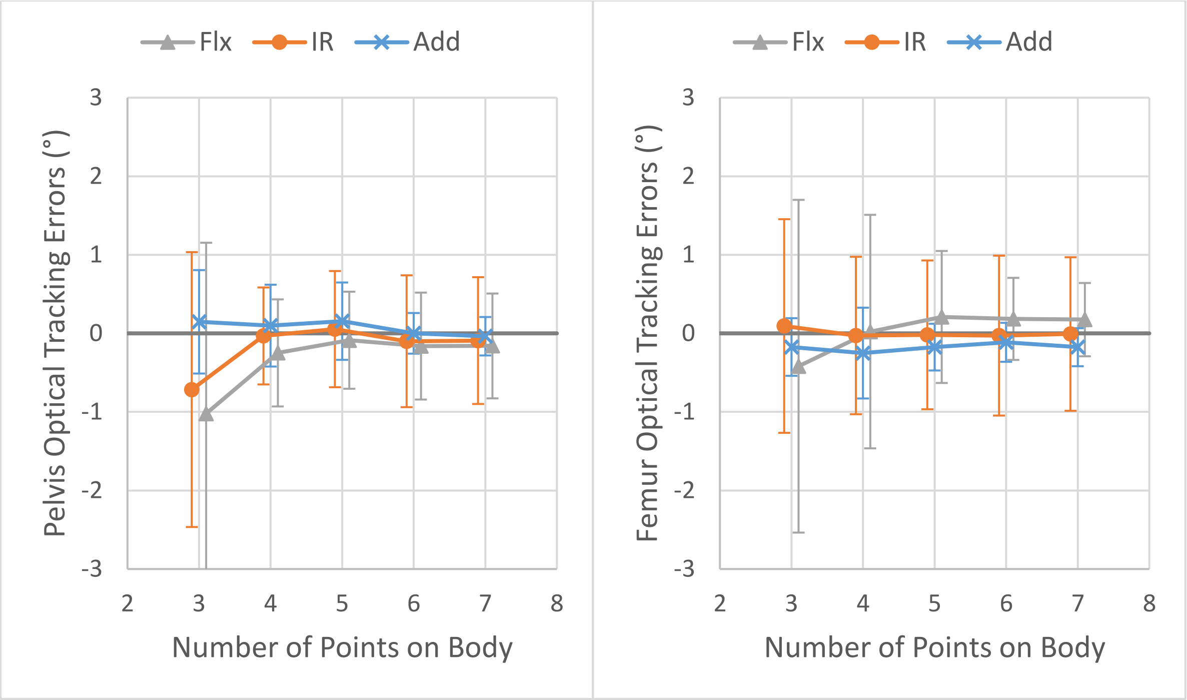

The mean error of the optical tracking system (n=8 specimens, with standard deviation) calculated by comparing an intact specimen digitised in the ISB coordinate system to itself (by comparing 3 or more repeats). The mean errors are shown for both the pelvis (left) and femur (right) in terms of flexion (Flx), internal rotation (IR) and adduction (Add). It can be seen that accuracy and/or precision of the measurement system improved until 6 or more data points had been digitised.
