# Supplementary material for: In vitro hip testing in the International Society of Biomechanics coordinate system
Source: J Biomech. 2016 Dec 8;49(16):4154–8. doi: 10.1016/j.jbiomech.2016.10.036 (PMC5352732; doi:10.1016/j.jbiomech.2016.10.036)
Supplement: Supplementary file 3 — Supplementary material [file mmc3.pdf]

Pelvic drilling guide

Step 1) The anterior superior iliac spines (ASISs) are first located in the bottom hole and slot (figure S1).

Step 2) The pelvis is then rotated until the posterior superior iliac spines (PSISs) can be visualised through the top slot.

Step 3) Holes representing the ISB X and Z axes can then be drilled into the pelvis using the guide.

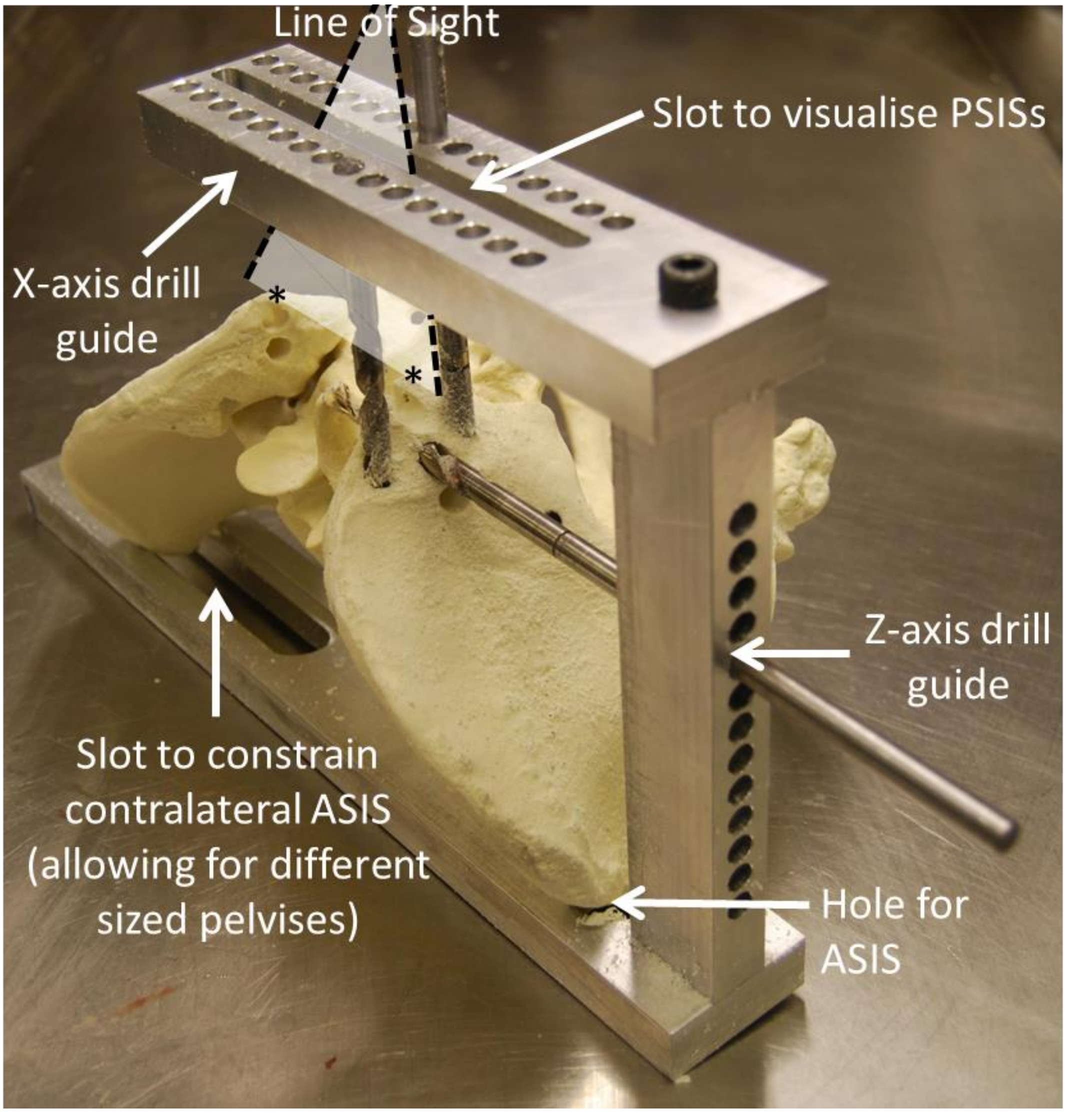

Figure S1: Pelvis drilling guide (femora not shown for clarity).

Femoral drilling guide

Step 1) The femoral head is held in place relative to the drilling guide. This can be done by first potting the pelvis, and then by fixing the pelvis pot to the drilling guide, as shown in figures S2-4, or by supporting the femoral head directly on the guide.

Step 2) The femoral epicondyles are then clamped at the same height between two plates to set neutral internal/external rotation by screwing a small screw into each epicondyles, placing on a horizontal surface and tightening the clamping bolts (figure S2).

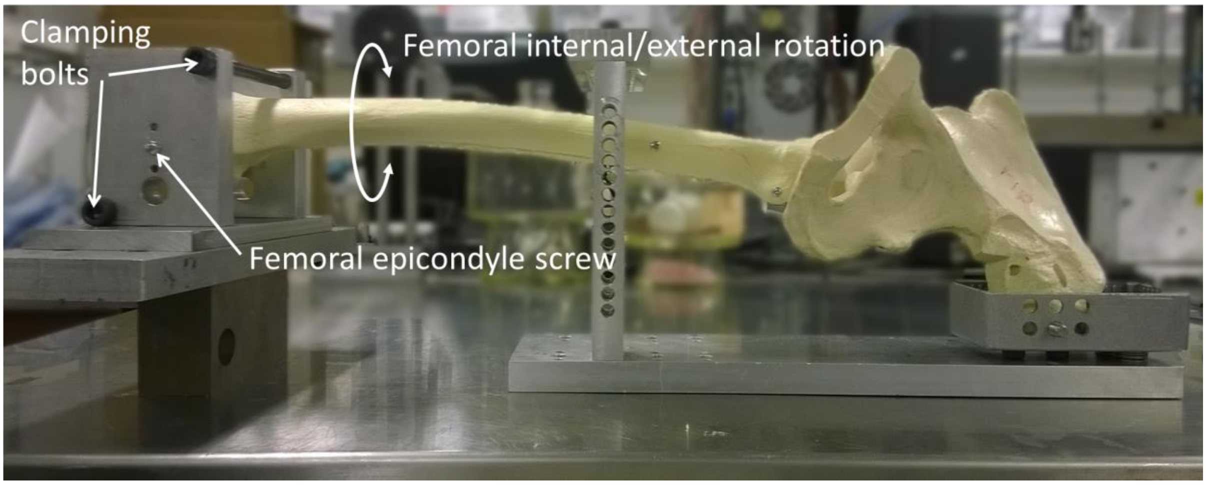

Figure S2: Clamping the epicondyles at the same height ensures neutral internal/external rotation of the femur

Step 3) The femoral y-axis is aligned with the drilling guide in the sagittal plane by raising or lowering the epicondyle clamping plates (a movement akin to hip flexion/extension) using scrap material as supports. This can be achieved visually by pulling a string taut between the estimated femoral head centre, and the femoral epicondyle screw and aligning the string parallel to the base of the drilling guide (figure S3). Alternatively, the height of the estimated femoral head centre from the base of the drilling guide can be measured and the femoral epicondyle height adjusted to match this value. Note that it is important not to raise one plate above the other so that neutral rotation about the femoral y-axis is maintained.

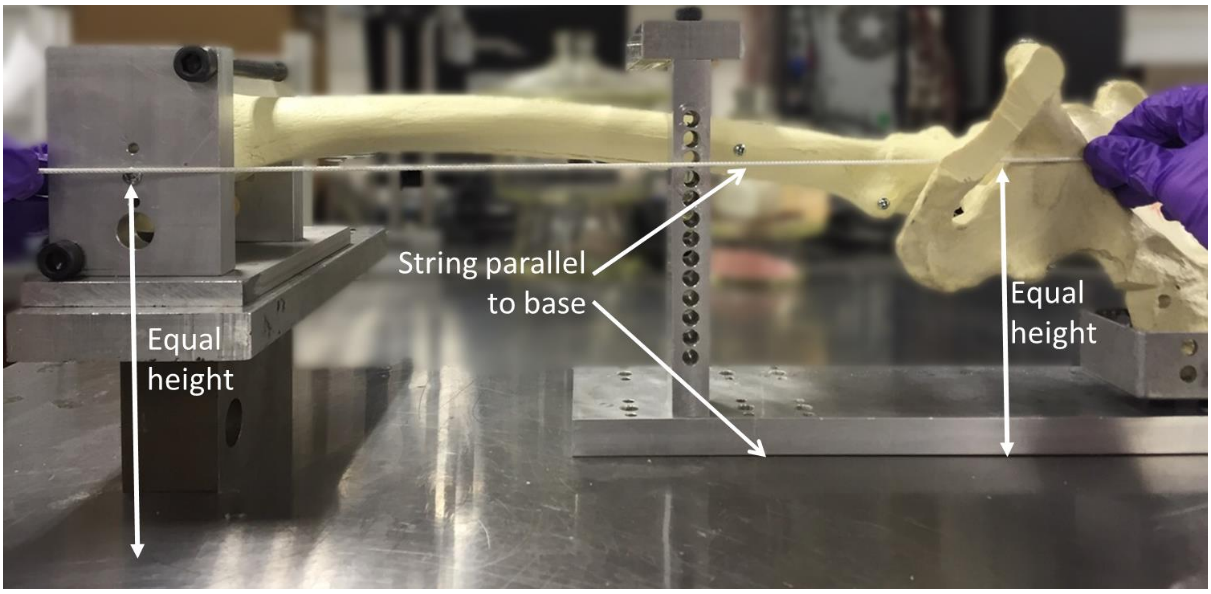

Figure S3: The epicondyle clamps are raised and lowered to align the femoral y-axis to the drilling jig in the sagittal plane

Step 4) The femoral y-axis is then aligned with the drilling guide in the coronal plane. To do this, a screw can be inserted at the midpoint of the femoral epicondyles and another piece of string pulled taut between the midpoint screw and the estimated femoral head centre. The epicondyle clamps are moved medially/laterally (akin to hip ab/adduction) until the string is parallel to the base of the drilling guide in the coronal plane (figure S4).

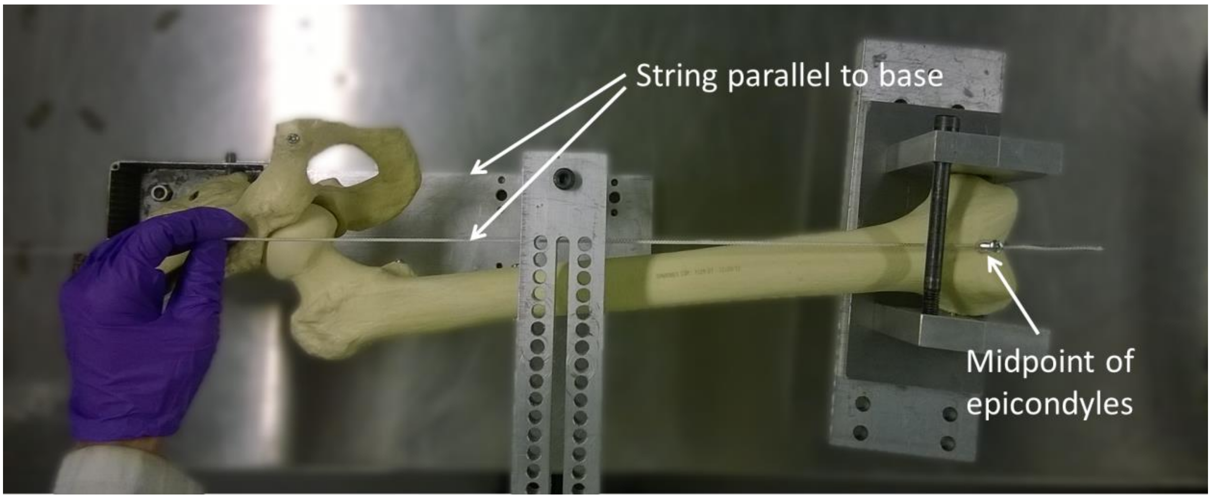

Figure S4: The epicondyle clamps are moved medially/laterally to align the femoral y-axis to the drilling jig in the coronal plane

Step 5) x-and z- axis reference holes are drilled into the bone using the guides. It can help to fix the drilling guide to the work bench using a g-clamp.

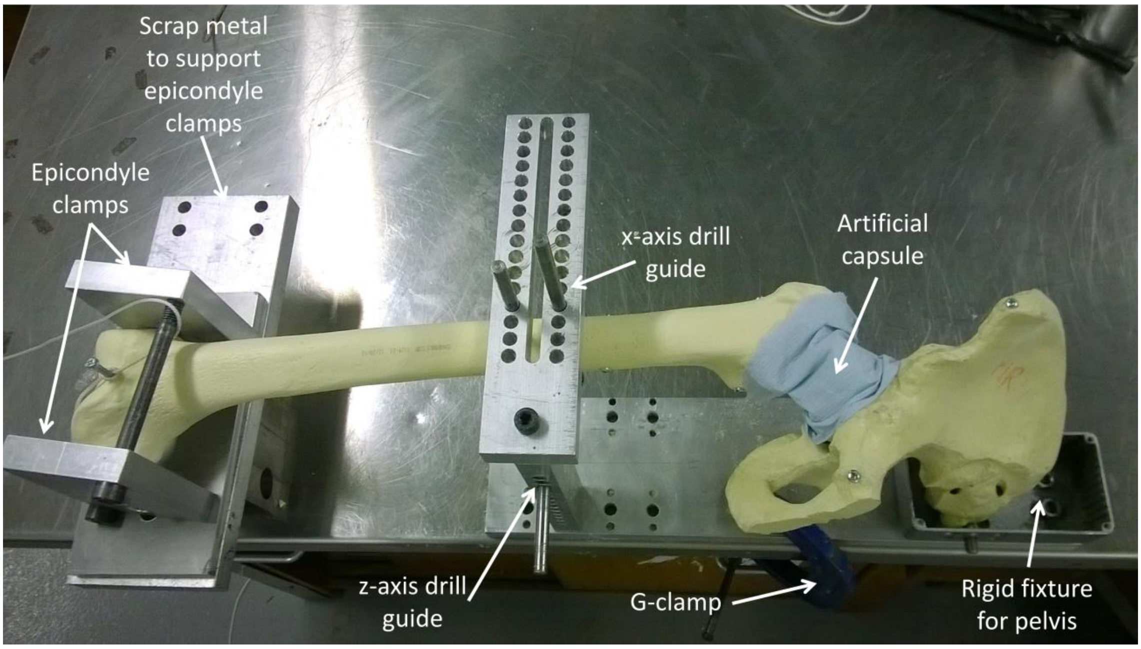

Figure S5: Using the drilling guide. The artificial capsule described in the manuscript is also shown.

Palpating the Femoral Head Centre

When the femoral head is not visible (for example when the hip joint capsule is intact) then the femoral head centre can be estimated by palpation. In the sagittal plane (figure S3), we estimate the femoral head centre as 5-10mm anterior of the posterior insertion of the transverse acetabular ligament into the acetabular rim, as shown in figure S5. When estimating the femoral head centre in the coronal plane, we approximate this to the iliopsoas groove medial to the anterior inferior iliac spine as shown in figure S6. Small inaccuracies caused by these approximations do not greatly affect the angular orientation of the femur to the drilling guides due to the long length of the femur (Figure S7 below and 6 in the manuscript).

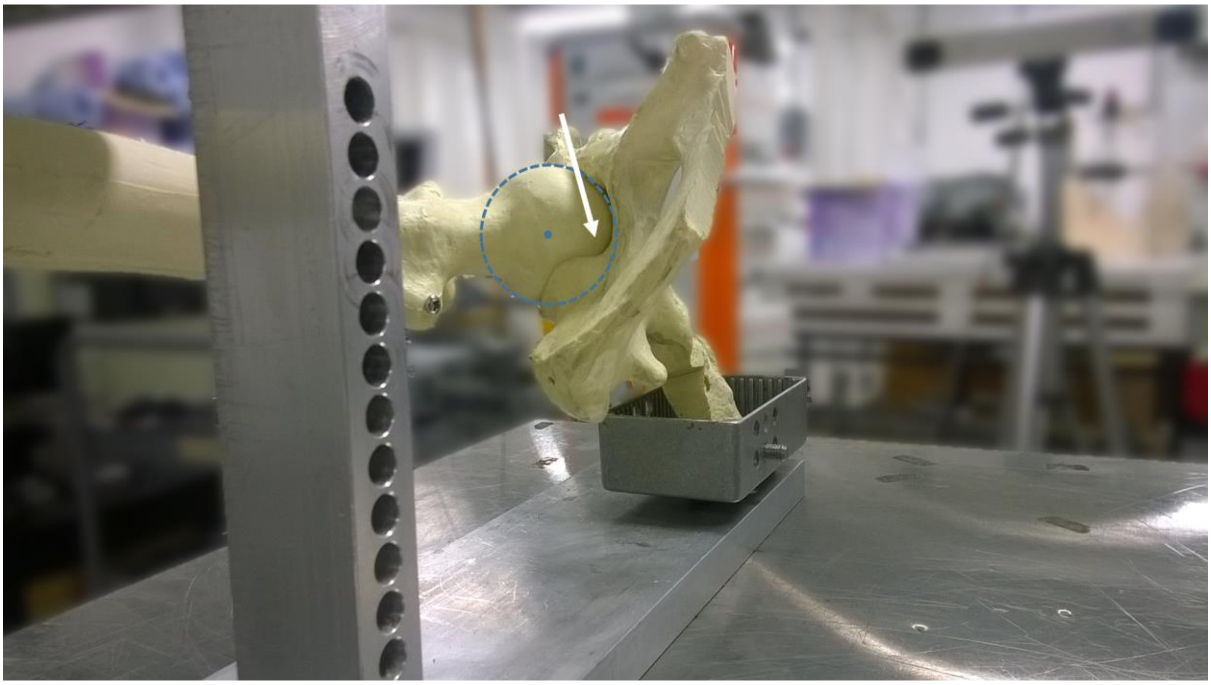

Figure S5 The femoral head centre's (blue dot in dashed circle) height in the sagittal plane can be approximated from the posterior insertion of the transverse acetabular ligament (white arrow).

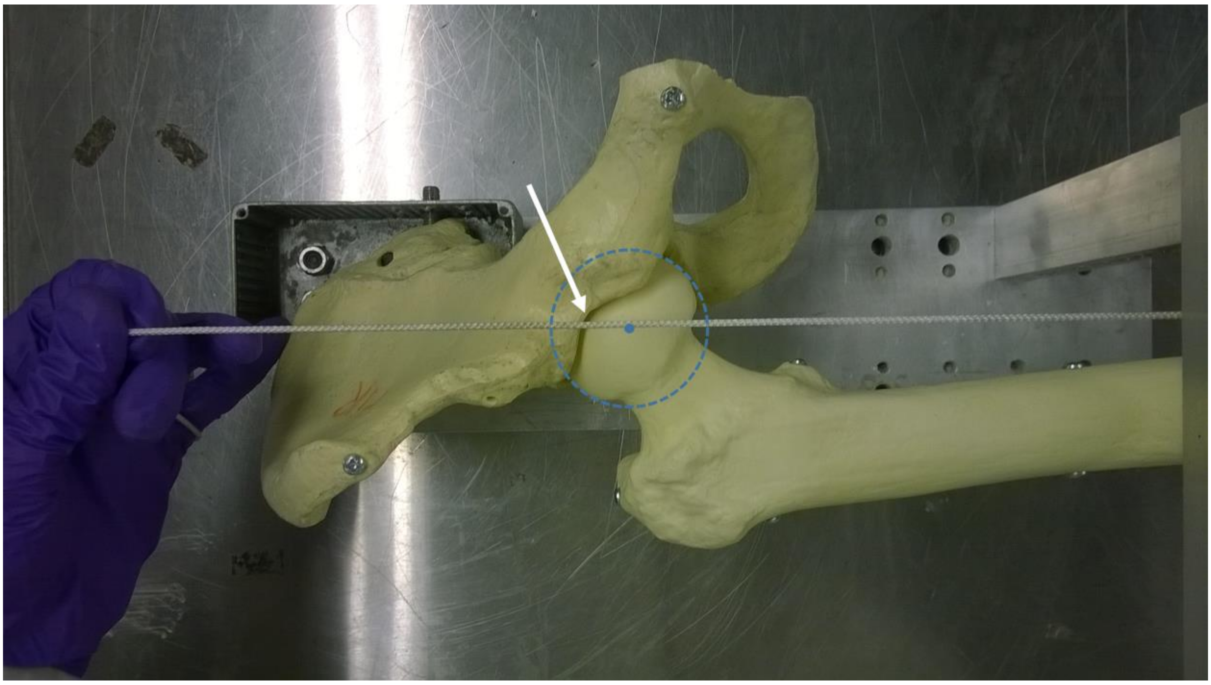

Figure S6 The femoral head centre's (blue dot in dashed circle) mediolateral position in the coronal plane can be approximated from the groove for the iliopsoas muscle (white arrow).

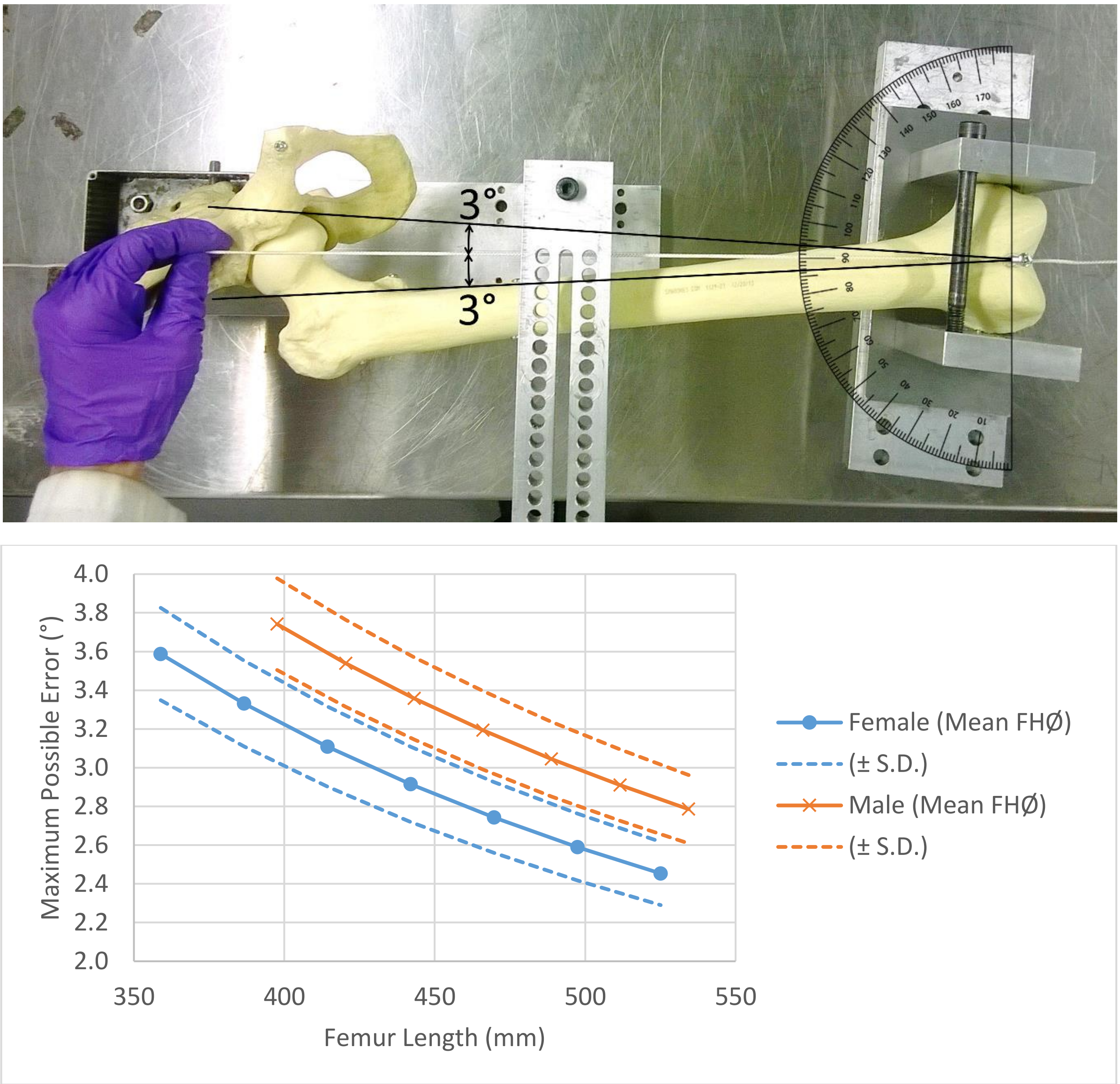

Figure S7 The maximum possible misalignment caused when estimating the femoral head centre provided that any point on the femoral head can be located/palpated. The x-axis values range from -3 to +3 standard deviations from the mean femur length reported for male (466 mm) and female (442 mm) femora (Yoshioka et al., 1987). The solid lines are the mean femoral head diameter (FHØ), and the dashed lines ± one standard deviation (S.D.) from this mean reported for male (52 ± 3.3 mm) and female (45 ± 3.0 mm) femora (Yoshioka et al., 1987). It can be seen that a shorter shaft, or a larger head, increase the maximum possible error, however, for all possible combinations (including an unlikely combination of large head and short femur) the estimated maximum error is estimated to be less than 4°. The visual representation of this calculation from the main manuscript (Figure 6) is also shown.

References

Yoshioka, Y., Siu, D., Cooke, T.D.V., 1987. The anatomy and functional axes of the femur. J Bone Joint Surg Am 69, 873-880.
